# Diverse roles of SARS-CoV-2 Spike and Nucleocapsid proteins in EndMT stimulation through the TGF-β-MRTF axis inhibited by aspirin

**DOI:** 10.1186/s12964-024-01665-z

**Published:** 2024-05-28

**Authors:** Wojciech M. Ciszewski, Lucyna A. Woźniak, Katarzyna Sobierajska

**Affiliations:** 1 Department of Molecular Cell Mechanisms, Medical University of Lodz, Mazowiecka Str. 6/8, Lodz, 92- 215 Poland; 2 Department of Structural Biology, Medical University of Lodz, Żeligowskiego Str. 7/9, Lodz, 90-752 Poland

**Keywords:** SARS-CoV-2, Fibrosis, EndMT, TGF-β, MRTFs, TLR4, ACE2, Aspirin

## Abstract

**Background:**

The SARS-CoV-2 virus causes severe COVID-19 in one-fifth of patients. In addition to high mortality, infection may induce respiratory failure and cardiovascular complications associated with inflammation. Acute or prolonged inflammation results in organ fibrosis, the cause of which might be endothelial disorders arising during the endothelial-mesenchymal transition (EndMT).

**Methods:**

HUVECs and HMEC-1 cells were stimulated with SARS-CoV-2 S (Spike) and N (Nucleocapsid) proteins, and EndMT induction was evaluated by studying specific protein markers via Western blotting. Wound healing and tube formation assays were employed to assess the potential of SARS-CoV-2 to stimulate changes in cell behaviour. MRTF nuclear translocation, ROS generation, TLR4 inhibitors, TGF-β-neutralizing antibodies, and inhibitors of the TGF-β-dependent pathway were used to investigate the role of the TGF-β-MRTF signalling axis in SARS-CoV-2-dependent EndMT stimulation.

**Results:**

Both viral proteins stimulate myofibroblast trans-differentiation. However, the N protein is more effective at EndMT induction. The TGF-β-MRTF pathway plays a critical role in this process. The N protein preferentially favours action through TGF-β2, whose secretion is induced through TLR4-ROS action. TGF-β2 stimulates MRTF-A and MRTF-B nuclear translocation and strongly regulates EndMT. In contrast, the Spike protein stimulates TGF-β1 secretion as a result of ACE2 downregulation. TGF-β1 induces only MRTF-B, which, in turn, weakly regulates EndMT. Furthermore, aspirin, a common nonsteroidal anti-inflammatory drug, might prevent and reverse SARS-CoV-2-dependent EndMT induction through TGF-β-MRTF pathway deregulation.

**Conclusion:**

The reported study revealed that SARS-CoV-2 infection induces EndMT. Moreover, it was demonstrated for the first time at the molecular level that the intensity of the EndMT triggered by SARS-CoV-2 infection may vary and depend on the viral protein involved. The N protein acts through TLR4-ROS-TGF-β2-MRTF-A/B, whereas the S protein acts through ACE2-TGF-β1-MRTF-B. Furthermore, we identified aspirin as a potential anti-fibrotic drug for treating patients with SARS-CoV-2 infection.

**Supplementary Information:**

The online version contains supplementary material available at 10.1186/s12964-024-01665-z.

## Introduction

Severe acute respiratory syndrome coronavirus-2 (SARS-CoV-2), a novel coronavirus, caused the outbreak of the coronavirus disease 2019 (COVID-19) pandemic, which is characterized by high contagiousness and mortality [1]. As a result of infection, almost 700 million cases were noted, and 7 million patients died worldwide in a little over three years (March 2020 - June 2023) [2]. The development of effective vaccines and mass vaccination, especially in highly developed countries, has caused a decrease in the number of deaths [3]. However, rapidly mutating viruses can still cause infections even after vaccination [4]. The course of the disease is still not well described and is challenging to predict, even in the vaccinated population. Similarly, the likelihood of complications in patients with COVID-19 is still limited [5].

Despite lower mortality in a highly vaccinated population, one of the significant problems observed in patients is tissue fibrosis of numerous organs due to SARS-CoV-2-induced inflammation [6]. The location of fibrotic changes and the degree of organ damage are crucial for the prognosis and quality of patient life spam. Unfortunately, the available data indicate that fibrosis may occur after severe, moderate, or mild COVID-19 [7]. It involves pulmonary, kidney, intestinal, and brain changes but can also develop in other tissues [8, 9]. It has been shown that high probability changes in the tissue might be correlated with their high vascularization, i.e., ECs contribute to more than 30% of all cells in the lungs [9] but also to the whole type of blood vessel, which is always covered with a layer of connected endothelial cells.

The crucial process inducing pathological fibrosis in endothelial-mesenchymal transition (EndMT) is based on the high plasticity of endothelial cells [10, 11]. This phenomenon is manifested by the loss of endothelial cell markers (claudin, occludin, E-cadherin and VE-cadherin) and disabled endothelial function together with the gaining of mesenchymal markers (vimentin or N-cadherin) and acquisition myofibroblast behaviour. Trans-differentiated cells secrete metalloproteinases and extracellular proteins (collagen I) that cause extracellular matrix (ECM) reorganization, favouring and facilitating cell migration [11–13]. The specific EndMT modification was observed in histopathological sections from the lungs of SARS-CoV-2-infected patients [14–16].

It has been shown that SARS-CoV-2 enters cells via angiotensin-converting enzyme 2 (ACE2) receptors, which are widely expressed on the endothelial and epithelial cell surface [17]. Under normal conditions, ACE2 converts angiotensin II (Ang II) to Ang-(1–7), and blocks tumour growth factor (TGF-β) synthesis. However, during SARS-CoV-2 infection, ACE2 is internalized, and as a result, its surface level decreases, stimulating TGF-β1 upregulation [18]. Because other members of the TGF-β family can induce cellular trans-differentiation, including endothelial-mesenchymal transition [19], its upregulation as a result of the interaction of SARS-CoV-2 with ACE2 might induce fibrosis pathways, stimulate inflammation by increasing cytokine secretion, and expose tissue for injury. Therefore, the endothelium has become a source of interest and might play a crucial role in fibrosis development during post-COVID-19 conditions, which is better known as long-term COVID-19 [19, 20].

To date, a few auto-inflammatory drugs, such as hydroxychloroquine, arbidol, redeliver, and favipiravir, have been approved to block the development of inflammation during the course of COVID-19 [10–13]. However, the above results demonstrated their limited efficacy in inhibiting post-COVID-19 fibrotic disorders. Therefore, there is an urgent need to understand the molecular mechanisms of SARS-CoV-2-dependent fibrosis induction to advance the development of effective drugs to prevent post-COVID-19 complications [21, 22]. In our studies, we focused on understanding the role of the molecular pathways involved in EndMT induction via two SARS-CoV-2 proteins, the Spike protein (S protein) and the Nucleocapsid protein (N protein), which are believed to play a significant role in COVID-19-induced fibrotic disorders.

## Materials and methods

### Cell lines

We used HUVECs (isolated by a standard procedure in our laboratory as described previously [23]), and Human Microvascular Endothelial Cells (HMEC-1; a gift from Kathryn Kellar, Centers for Disease Control and Prevention, Atlanta, GA). The cells were maintained in MCDB 131 medium supplemented with 10% (v/v) FBS, streptomycin (100 µg/mL), penicillin (100 units/mL), glutamine (2 mM), epidermal growth factor (EGF; 10 ng/mL), and hydrocortisone (1 µg/mL; HMEC-1 only) at 37 °C, in a humidified atmosphere with 5% CO_2_. The cells were routinely tested and confirmed to be mycoplasma-free. The experiments were conducted within passages 1–5 of HUVEC and 16–20 of HMEC-1 cells. All media and supplements were obtained from Life Technologies (Paisley, UK), except for EGF and hydrocortisone, which were obtained from Sigma‒Aldrich (Darmstadt, Germany).

### Cell stimulation and treatments

Recombinant human SARS‑CoV‑2 proteins, 2019-nCoV S1 Protein (32-190005), and 2019-nCoV Nucleocapsid Protein (32-190002) were purchased from Abeomics (San Diego, CA, USA); TGF‑β2 was purchased from R&D Systems (Minneapolis, MN, USA); and C3 Transferase Protein, (C3, bacterial recombinant; CT03) was obtained from Cytoskeleton, Inc. (Denver, CO, USA). All proteins were reconstituted according to the manufacturer’s instructions. A Smad3 inhibitor (SIS3) was purchased from Merck Life Science (Calbiochem, Darmstadt, Germany), and aspirin (ASA) and TAK-242 were obtained from Sigma Aldrich (Darmstadt, Germany). Anti-TGF-β1 (TB21) and anti-TGF-β2 (2G7) antibodies were purchased from Thermo Fisher Scientific (Rockford, IL, USA). All proteins and compounds were aliquoted and stored at -70 °C until use.

Cells were treated with 0.5 µg/mL S or N SARS‑CoV‑2 proteins for 48 h prior to further analysis. The concentration of SARS-CoV-2 proteins was non-toxic for studied cells. As a positive control for EndMT induction, the cells were treated with 10 ng/mL TGF-β2 for 48 h, as previously described [24]. In studies with SIS3, TAK-242, and C3 inhibitors, the cells were treated with 3 µM, 5 µM, and 10 µM, respectively, for 1 h prior to cell stimulation with SARS‑CoV‑2 proteins for 48 h. ASA (2.5 mM) was added to the cells 24 h before or 24 h after stimulation with the viral proteins. The ASA at the utilized dose did not affect cell viability. To neutralize TGF-β, the cells were incubated with 5 µg/mL anti-TGF-β1 or anti-TGF-β2 for 1 h. Then, the cells were stimulated with SARS‑CoV‑2 proteins, as indicated previously.

### Western blotting

The procedure was performed as previously described [25]. Briefly, total cell lysates, cell surface fractions, or nuclear fractions were prepared from exponentially growing endothelial cells. Cells were lysed in M-PER Mammalian Protein Extraction Reagent (Thermo Scientific, Rockford, IL, USA) with cOmplete Mini, EDA-free Protease Inhibitor Cocktail (Roche, Mannheim, Germany), the cell surface fraction was isolated with NE-PER Nuclear and Cytoplasmic Extraction Reagents (Thermo Scientific, Rockford, IL, USA), and the nuclear fraction was isolated with a Cell Surface Protein Isolation Kit (Thermo Scientific, Rockford, IL, USA) according to the manufacturer’s instruction. Next, the supernatants were aliquoted and stored at − 70 °C until use. Protein levels were evaluated with a BCA Protein Assay Kit (Thermo Scientific, Rockford, IL, USA), and 30 µg of each lysate was separated on 4–12% Bold Bis-Tis Plus gradient gels (Thermo Scientific, Carlsbad, CA, USA). The proteins were electroblotted onto nitrocellulose membranes (Amersham Protean 0.45 *µ*m, GE Healthcare, Bensheim, Germany), blocked in SuperBlock (PBS) buffer (Thermo Scientific Rockford, IL, USA) and incubated with primary antibodies overnight at 4 °C. Then, the membrane was washed with 0.05% Tween in Tris-Buffered Saline (TBST) and incubated with secondary antibodies conjugated with horseradish peroxidase (Dako, Ely, UK) for 1 h at room temperature. Then, antibody binding was visualized by chemiluminescence using ECL Western Blotting Substrate (SuperSignal West Pico Plus, Thermo Scientific, Rockford, IL, USA) and CL-XPosure Film (Agfa Healthcare, Mortsel, Belgium). The films were scanned using an HP Scanjet G4050 scanner (Hewlett Packard, Palo Alto, C, USA), and the bands were quantified by ImageJ software v 1.47 (Bethesda, MD, USA). The area for each protein peak was determined to quantify the immunofluorescence intensity after a background subtraction. If necessary, the membrane was stripped in Restore PLUS Western Blot Stripping Buffer (Thermo Scientific, Rockford, IL, USA) and re-probed as described above. Protein levels were normalized using an appropriate loading control. The primary antibodies used were against Snail, Slug, vimentin, N-cadherin, E-cadherin, claudin (Cell Signaling Technology, Inc., Beverly, MA, USA), His3, caldesmon, tropomyosin, *α*-SMA (Sigma Aldrich (Darmstadt, Germany), TLR4 LYVE-1 (Abcam, Cambridge, UK), MRTF-A, MRTF-B and GAPDH (Santa Cruz Biotechnology, Dallas, TX, USA).

### Tube formation assay

Capillary-like tube formation was evaluated in 12-well plates precoated with Matrigel as described previously [23]. Briefly, cells (4 × 10^4^) were seeded on plates, and after 8 h of incubation, the capillary-like structures were observed and imaged with an EVOS FLoid Cell Imaging Station (Thermo Fisher Scientific, Bothell, WA, USA). The total length of the capillaries was analysed with ImageJ software v 1.47 by the plugin Angiogenesis Analyser.

### Wound healing

Cells were seeded in 12-well plates and, after reaching a monolayer, were scratched across the well with a 200 µL pipette tip. Next, the cells were rinsed twice with medium and allowed to migrate in the incubator. Images were captured immediately after scratching (time = 0) and eight hours later, with an EVOS FLoid Cell Imaging Station (Thermo Fisher Scientific, Bothell, WA, USA). A minimum of four randomly selected fields of view of the denuded area were captured, and the cell migration was quantified with ImageJ software v 1.47 by the macro Wound Healing Tool.

### Fluorescence microscopy

The cells were placed on sterile glass microscope slides and cultured at 37 °C in a humidified atmosphere of 5% CO_2_. After appropriate treatment, the cells were fixed with 4% formaldehyde in PHEM buffer (60 mM Pipes, pH 6.9, containing 25 mM HEPES, 10 mM EGTA, and 4 mM MgCl_2_) supplemented with protease inhibitors (cOmplete™, EDTA-free Protease Inhibitor Cocktail), and labelled as described previously [24]. The images were visualized with the EVOS FLoid Cell Imaging Station (Thermo Fisher Scientific, Bothell, WA, USA), and the fluorescence intensity was measured with ImageJ v 1.47 using the encircled region of interest (ROI).

### ROS generation

Intracellular ROS generated in the cells were measured using H2DCFDA-AM (Life Technologies), a cell-permeable dye that measures reactive oxygen species (ROS) activity within cells. Briefly, the cells were seeded on 96-well microplates and treated as indicated previously. Next, the medium was replaced with fresh medium, and H2DCFH-DA was added to the culture medium (5 µM final concentration). After 30 min of incubation in the dark at 37 °C, the cells were washed twice with PBS, and the fluorescence was measured using a SpectraMax M5 microplate reader (Molecular Devices, San Jose, CA, USA) with maximum excitation and emission wavelengths of 495 and 525 nm, respectively. The results are shown as the ratio of fluorescence to fluorescence emitted by the unstimulated control cells.

### Statistical analysis

Each experiment was performed at least three times. Statistical significance was evaluated using Student’s t-test or one-way analysis of variance (ANOVA) followed by Tukey’s test with GraphPad Prism Software v 9.3. Differences between means were considered significant at *p* ≤ 0.05. The results are presented as the means ± standard errors.

## Results

### SARS-CoV-2 S protein and N protein induced EndMT in endothelial cells

Previous studies have suggested that the endothelium undergoes EndMT due to COVID-19 [14]. Since COVID-19 might be harmful to various organs, we used endothelial cells of different origins isolated from microvascular vessels (HMEC-1 cells) and the umbilical cord (HUVECs). To verify the assumption about a SARS-CoV-2-dependent EndMT induction, EndMT markers and behavioural analysis of endothelial cells stimulated with the N or S protein were performed. We employed TGF-β2 as a positive control for EndMT stimulation [23].

**Fig. 1:**
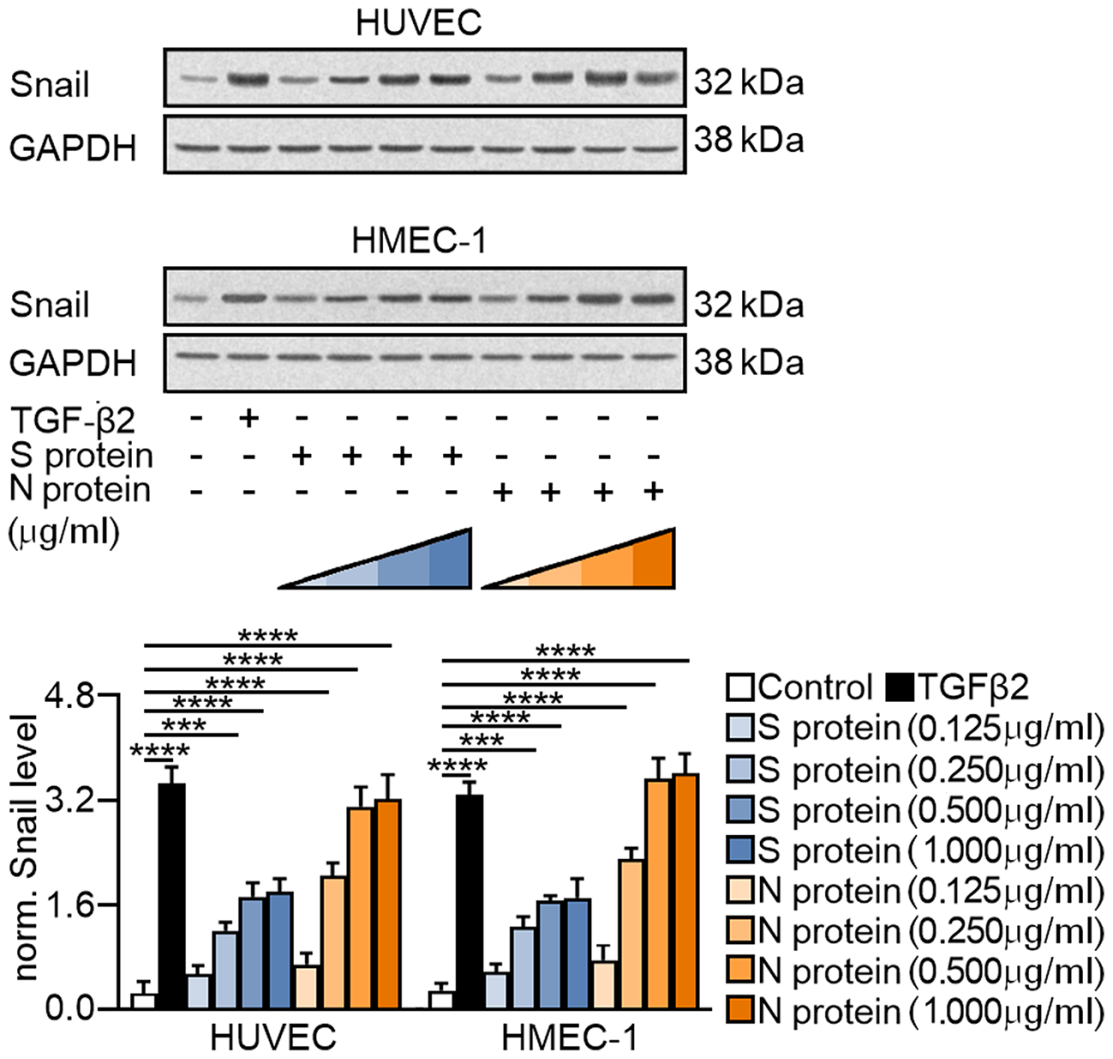
The SARS-CoV-2 S and N proteins stimulate the expression of Snail in a concentration-dependent manner in the endothelium. Endothelial cells (HUVECs or HMEC-1 cells) were treated with the SARS-CoV-2 S protein or N protein at the indicated concentrations for 48 h. As a positive control, cells were treated with 10 ng/mL TGF-β2 for 48 h. Snail protein levels were analysed by Western blot. Representative blots are shown. The protein level was normalized to GAPDH. The graphs display the means ± S.D. (*n* = 3). ***P < 0.001, ****P < 0.0001

First, we studied whether the SARS-CoV-2 proteins induce Snail, the critical transcription factor regulating EndMT. We observed that both SARS-CoV-2 proteins stimulated an increase in Snail in a concentration-dependent manner (Fig. 1). Moreover, it should be noted that the observed increase in the Snail protein level was more significant when the cells were treated with N protein. Based on the results of our analysis, we determined that 0.5 µg/mL was the optimal dose of the SARS-CoV-2 protein for stimulating endothelial cells, and this concentration was used in further studies.

**Fig. 2:**
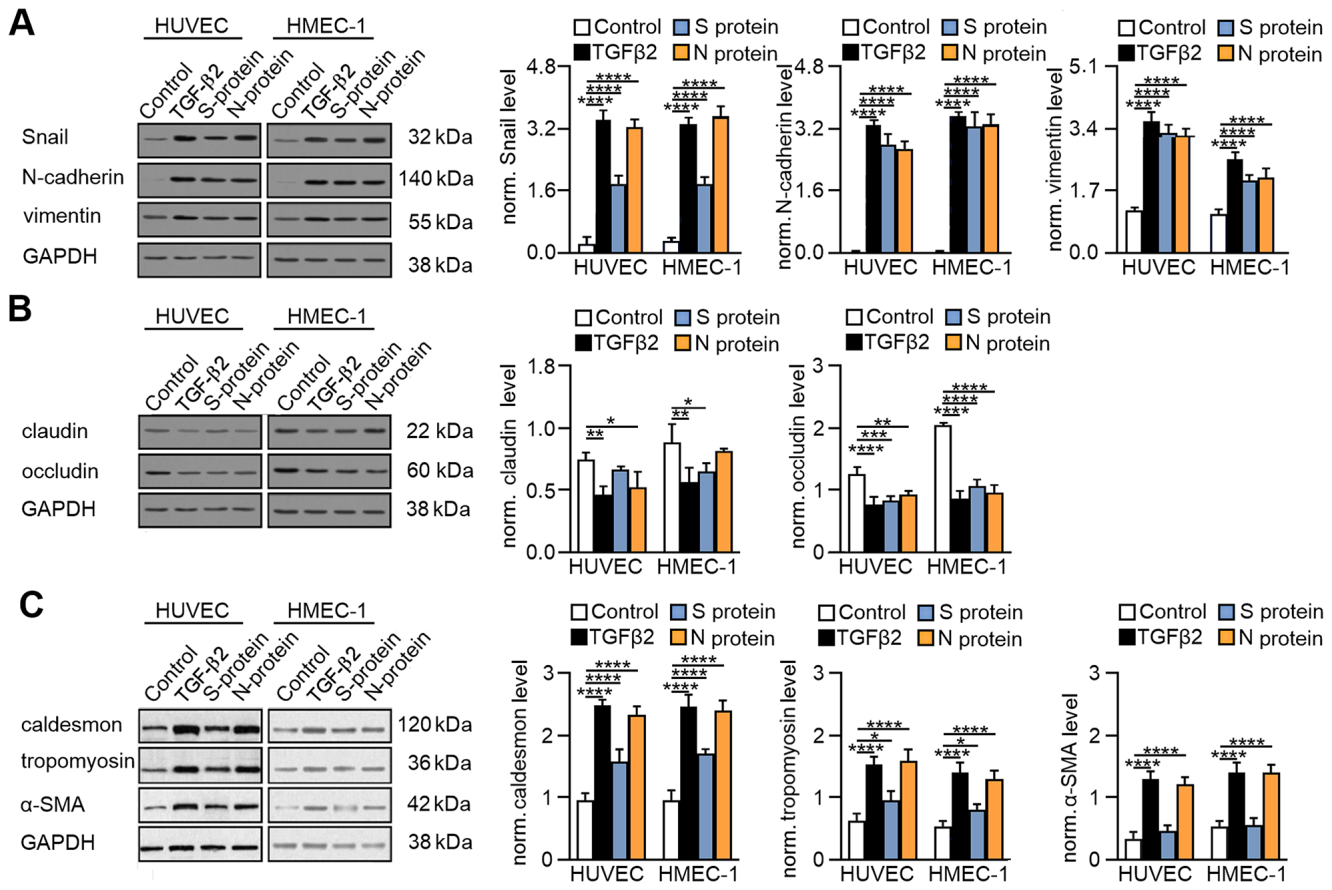
SARS-CoV-2 proteins induce EndMT through the modulation of specific endothelial and mesenchymal marker levels. Endothelial cells were treated with S protein (0.5 *µ*g/mL) or N protein (0.5 *µ*g/mL) for 48 h. Cells treated with 10 ng/mL TGF-β2 for 48 h were used as a positive control. (**A**) The levels of mesenchymal markers, (**B**) the levels of endothelial markers and (**C**) the levels of contraction protein markers were analysed by Western blot. Representative blots are shown. The protein level was normalized to GAPDH. The graphs display the means ± S.D. (*n* = 3). *P < 0.05, **P < 0.01, ***P < 0.001, ****P < 0.0001

Then, we analysed the effect of SARS-CoV-2 on EndMT stimulation. The analysis of mesenchymal markers revealed a strong upregulation of these markers in cells treated with SARS-CoV-2 proteins (Fig. 2A). Vimentin and N-cadherin protein levels significantly increased in cells to the level observed after the TGF-β2 stimulation. Similar results were observed for Snail level in cells treated with the N protein, whereas the S protein had a slightly lower effect on Snail stimulation (Fig. 2A). Simultaneously, we observed a decrease in endothelial marker levels in cells due to SARS‑CoV‑2 protein stimulation. The virus proteins caused a substantial decrease in the occludin protein level and a moderate reduction in the claudin protein level (Fig. 2B).

During myofibroblast formation, cells acquire contraction ability [14]. Thus, in the next step, we examined the levels of these proteins in endothelial cells stimulated with SARS-CoV-2 proteins. We observed that the N protein significantly increased the level of contractile proteins in the treated cells, which corresponded to the effect of TGF-β2 (Fig. 2C). Similar to the results obtained for mesenchymal markers, the impact of the S protein was lower but statistically significant (Fig. 2C).

**Fig. 3:**
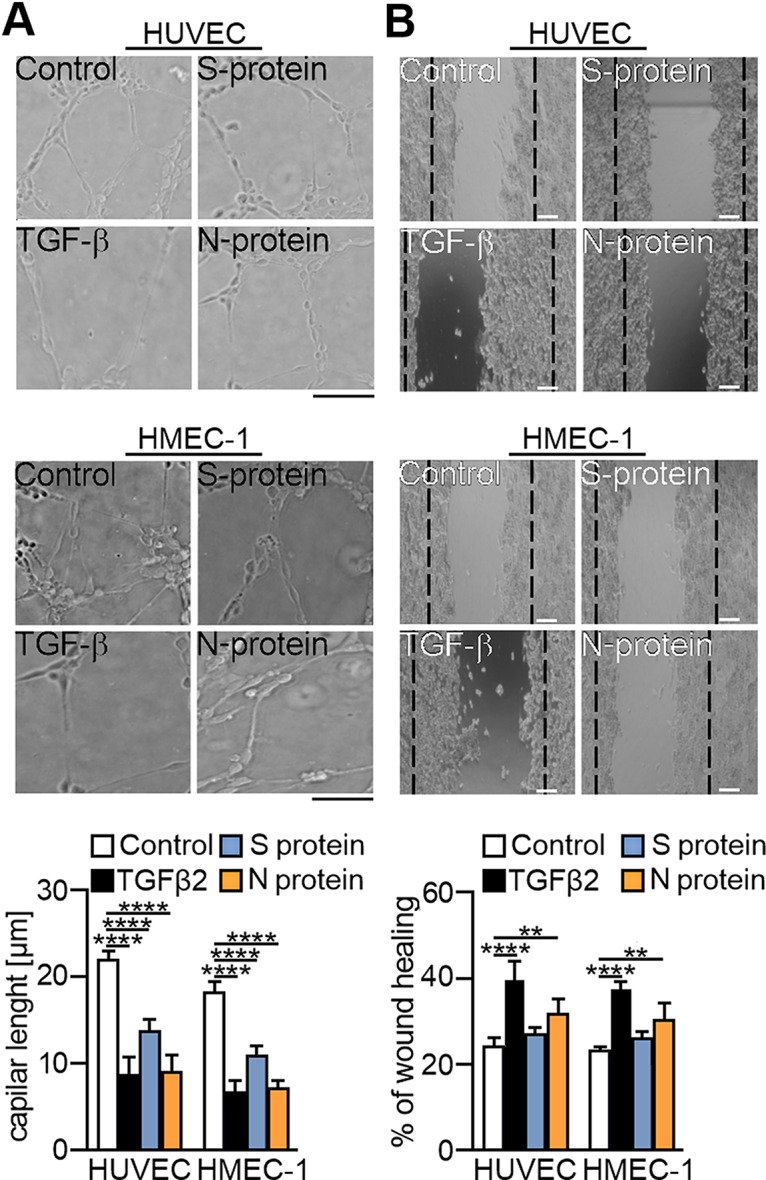
SARS-CoV-2 proteins stimulate changes in the behaviour of endothelial cells. Endothelial cells were treated with S protein (0.5 *µ*g/mL) or N protein (0.5 *µ*g/mL) for 48 h. Cells treated with 10 ng/mL TGF-β2 for 48 h were used as a positive control. (**A**) Capillary formation ability was tested by a tube formation assay, and the total length of the capillaries is shown in the graph. (**B**) Cell migration potential was analysed by a wound healing assay. Representative images are shown. Scale bars, 100 μm. The graphs display the means ± S.D. (*n* = 3). *P < 0.05, **P < 0.01, ****P < 0.0001

Furthermore, we verified whether changes in the protein levels of EndMT markers correspond to changes in the behaviour of endothelial cells. We analysed the effect of SARS-CoV-2 proteins on capillary-like structure formation, which is the ability that endothelial cells lose due to EndMT. We demonstrated that SARS‑CoV‑2 proteins decreased capillary formation (Fig. 3A). The S and N proteins caused a 40% and 60% reduction in capillary length, respectively. A similar effect being 60% was observed for TGF-β2 stimulation (Fig. 3A). Since transdifferentiation to mesenchymal cells is associated with gaining the migration ability, we analysed the influence of SARS-CoV-2 proteins on cell migration. We observed that the S protein and N protein stimulated cells to faster movement by 13% and 32%, respectively, while TGF-β2 had the most significant effect at 63%. (Fig. 3B).

### The SARS-CoV-2 S and N proteins modulated the secretion of TGF-β family members

**Fig. 4:**
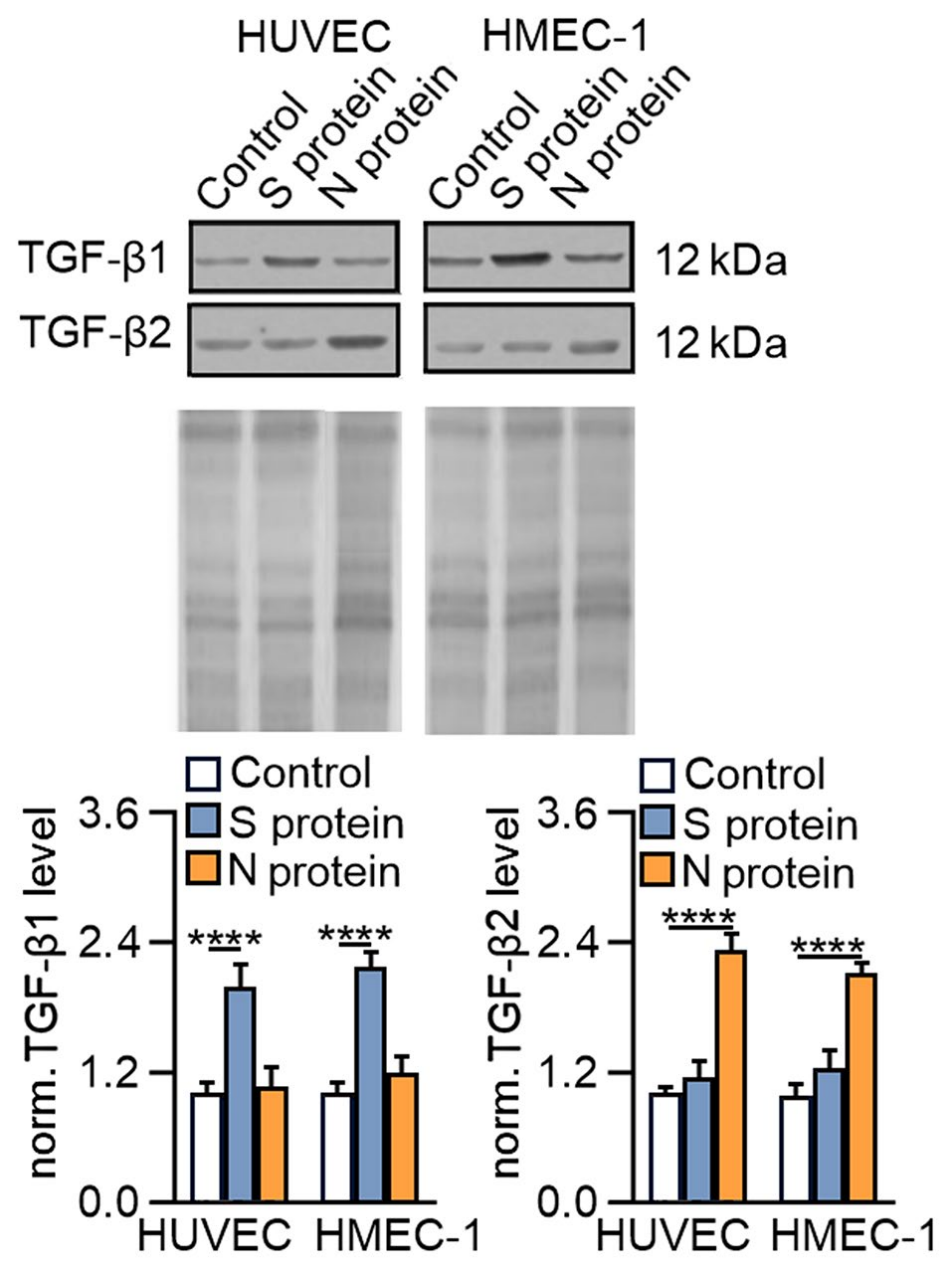
SARS-CoV-2 proteins stimulate TGF-β1 and TGF-β2 secretion in endothelial cells. Cells were treated with S protein (0.5 *µ*g/mL) or N protein (0.5 *µ*g/mL) for 48 h. Then, the levels of TGF-β1 and TGF-β2 were measured by Western blotting. Coomassie blue was used as the loading control. Representative blots are shown. The graphs display the means ± S.D. (*n* = 3). ****P < 0.0001

Since growth factors belonging to the TGF-β family are the most potent regulators of EndMT, we investigated whether stimulation of endothelial cells with SARS-CoV-2 proteins would modulate TGF-β secretion. We revealed that S protein-stimulated TGF-β1 secretion was around 2-fold higher than in control cells in both cell lines. The N protein caused only a slight but non-significant increase of TGF-β1, which was 1.09- and 1.25-fold more elevated in HUVECs and HMEC-1 cells, respectively, than in control cells. Interestingly, we observed a reverse effect on TGF-β2 secretion due to stimulation of cells with SARS-CoV-2 proteins. S protein treatment resulted in a non-significantly increase of TGF-β2, which was 1.13- and 1.25-fold higher than that in untreated HUVECs and HMEC-1 cells, respectively. In contrast, the N protein caused a more than 2.1-fold increase in TGF-β2 secretion in both studied endothelial cell lines (Fig. 4).

### The SARS-CoV-2 proteins stimulated the secretion of TGF-β via the ACE2 or TLR4–ROS pathway

**Fig. 5:**
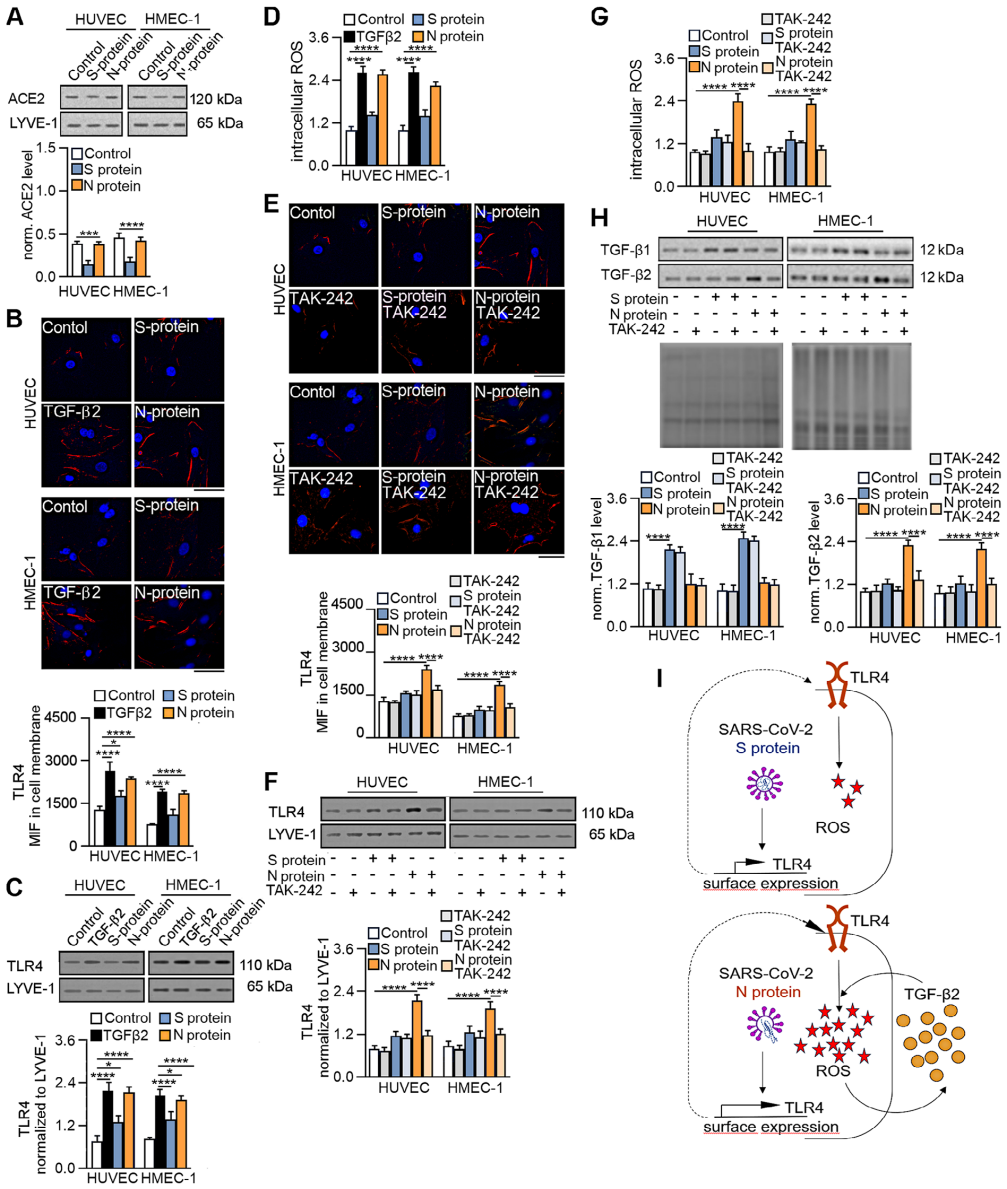
SARS-CoV-2 proteins stimulate TRL4 activation and ROS production in endothelial cells. Cells were treated with S protein (0.5 µg/mL) or N protein (0.5 µg/mL) for 48 h. Cells treated with 10 ng/mL TGF-β2 for 48 h were used as a positive control. Then, the ACE2 surface protein level was analysed by (**A**) Western blot, and the TRL4 surface protein level was analysed by (**B**) immunostaining and (**C**) Western blot. (**D**) Intracellular ROS levels were estimated in cells by H2DCFDA-AM dye. Next, the cells were treated with 1 µM TAK-242 for 2 h prior to stimulation with the S protein (0.5 µg/mL) or N protein (0.5 µg/mL) for 48 h. Then, the TRL4 surface protein level was analysed by (**E**) immunostaining and (**F**) Western blot analysis. (**G**) Intracellular ROS levels were estimated in cells by H2DCFDA-AM dye. (**H**) The levels of TGF-β1 and TGF-β2 were measured by Western blotting. Coomassie blue was used as the loading control. Representative images and blots are shown. The protein levels of ACE2 and TRL4 were normalized to LYVE-1. Scale bars, 100 μm. The graphs display the means ± S.D. (*n* = 3). *P < 0.05, ****P < 0.0001. (**I**) Schematic overview of the role of the SARS-CoV-2 S and N proteins in TLR4 activation and ROS production

With data showing that the N protein has a more substantial effect on EndMT than does the S protein and suggesting that this modulation is regulated by TGF-β2 secretion, we sought to investigate the underlying mechanism. First, we focused on the interaction of SARS-CoV-2 proteins with ACE2, which is known to be involved in the SARS-CoV-2 entry pathway [18]. We revealed that the SARS-CoV-2 S protein decreased the ACE2 surface protein level by 60%, and the N protein did not affect the ACE2 level (Fig. 5A).

Since we did not detect the effect of the N protein, we focused on another TLR4-dependent pathway that is believed to be involved in cell infection with the SARS-CoV-2 virus [26]. It has been proposed that TLR4 mediates the reactive oxygen species (ROS) signalling pathway [27]. Since ROS modulate TGF-β signalling through different pathways, we verified whether the N protein might stimulate EndMT through the TLR4-ROS-TGF-β axis. We observed that S protein treatment caused a slight increase in the TLR4 surface protein level, which was 1.4- and 1.7-fold higher than that in nontreated cells, as measured by immunostaining and WB, respectively (Fig. 5B and C). In contrast, N protein caused more than 2.1-fold and 2.8-fold increases in TLR4 protein levels, as measured by both methods (Fig. 5B and C). Next, we revealed that the N protein strongly stimulated ROS production, which was 2.5-fold higher in the N protein-treated cells than in the nontreated cells. At the same time, the S protein caused only a slight but non-significant increase in ROS production, which was 1.35-fold higher than that in the control (Fig. 5D).

Next, TAK-242, a TLR4 inhibitor, was used to confirm the involvement of TLR4 in SARS-CoV-2-dependent EndMT modulation. TAK-242 did not affect TLR4 protein levels in cells stimulated with the S protein. In comparison, in SARS-CoV-2 N-stimulated cells, TAK-242 decreased the TLR4 surface level by 70% (Fig. 5E and F). A similar effect of the TLR4 inhibitor was observed when the stimulation of ROS production by SARS-CoV-2 proteins was examined. We observed no effect or complete inhibition of ROS production in cells treated with the S or N protein, respectively (Fig. 5G). Then, we investigated whether TLR4 inhibition affects TGF-β secretion in SARS-CoV-2-stimulated cells. TAK-242 had no effect on TGF-β1 secretion but inhibited TGF-β2 secretion in N protein-stimulated cells (Fig. 5H). These results indicate that the SARS-CoV-2 S protein only slightly stimulates the TLR4-ROS pathway. On the other hand, the N protein strongly activates this pathway, which stimulates TGF-β2 secretion (Fig. 5I).

### SARS-CoV-2 proteins stimulate EndMT in a TGF-β-dependent manner

To verify that the observed effects of SARS-CoV-2 proteins on EndMT stimulation resulted from TGF-β secretion, we used TGF-β1- and TGF-β2-neutralizing antibodies. We observed that anti-TGF-β antibodies profoundly decreased the level of TGF-β secreted into the medium after SARS-CoV-2 stimulation (Fig. S1).

**Fig. 6:**
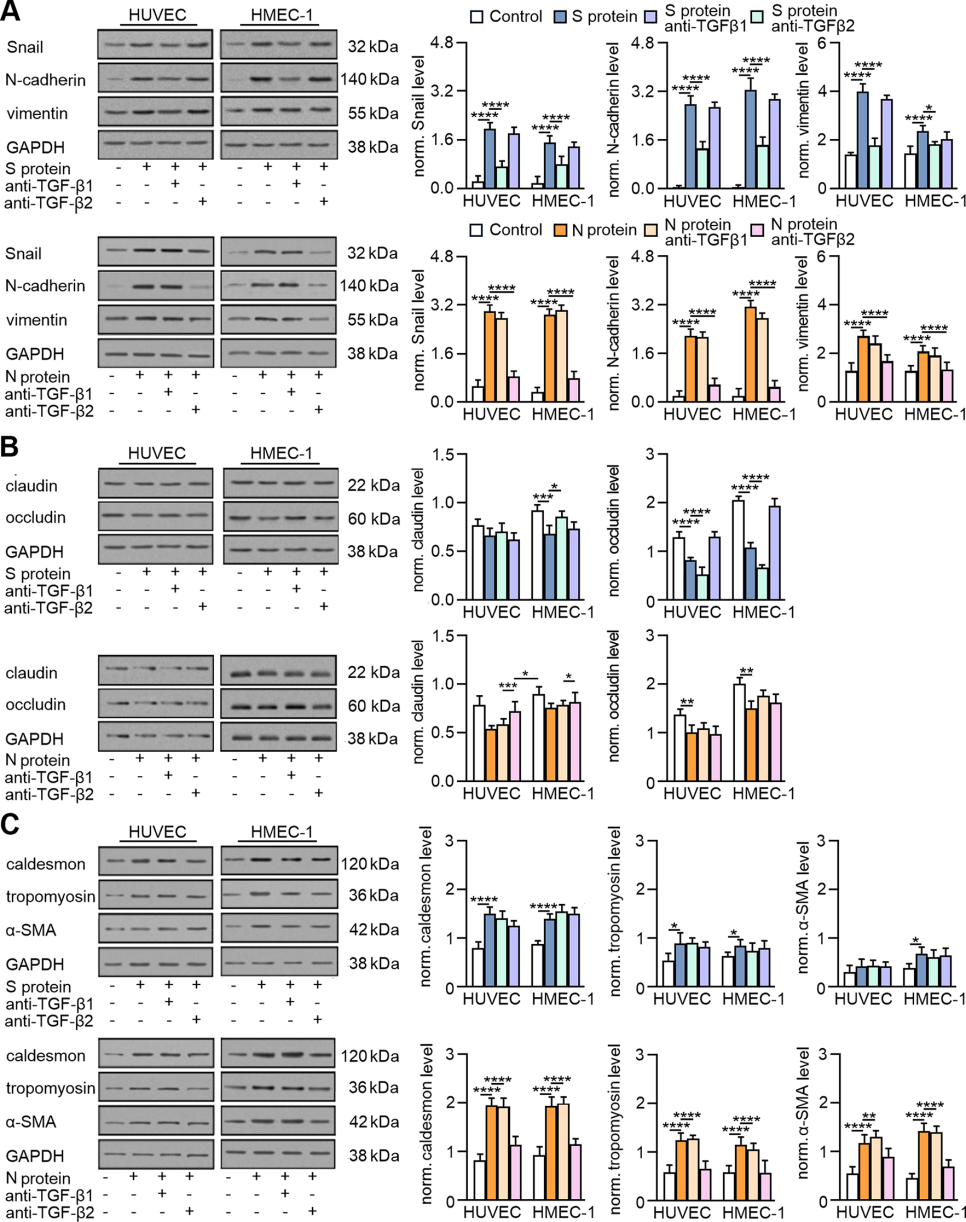
TGF-β2 is a critical modulator of EndMT in endothelial cells treated with SARS-CoV-2 proteins. Cells were treated with 5 µg/mL anti-TGF-β1 or anti-TGF-β2 antibodies for 1 h prior to stimulation with S protein (0.5 *µ*g/mL) or N protein (0.5 *µ*g/mL) for 48 h. (**A**) The levels of mesenchymal markers, (**B**) the levels of endothelial markers and (**C**) the levels of contraction protein markers were analysed by Western blot. Representative blots are shown. The protein level was normalized to GAPDH. The graphs display the means ± S.D. (*n* = 3). *P < 0.05, **P < 0.01, ****P < 0.0001

**Fig. 7:**
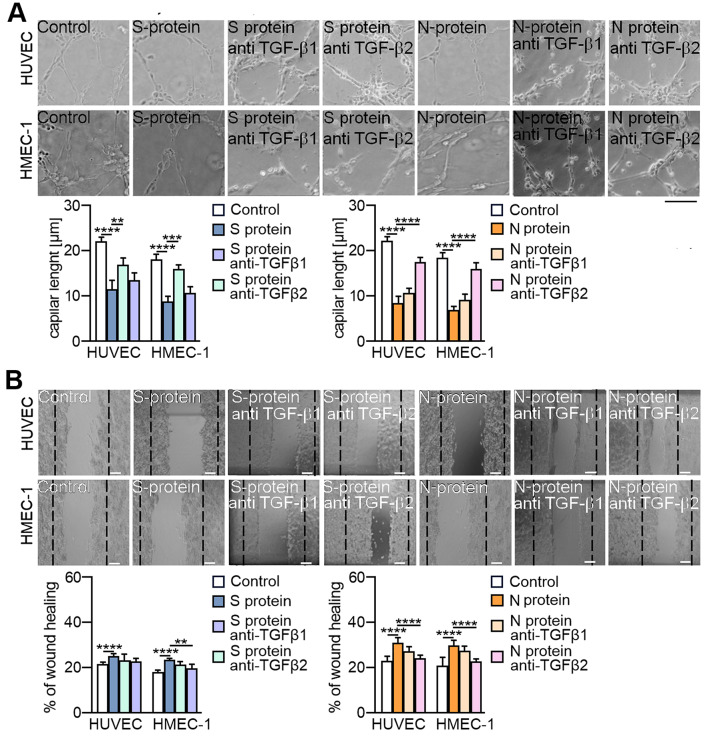
TGF-β2 is the main factor responsible for changes in the behaviour of endothelial cells treated with SARS-CoV-2 proteins. Cells were treated with 5 µg/mL anti-TGF-β1 or anti-TGF-β2 antibodies for 1 h prior to stimulation with S protein (0.5 *µ*g/mL) or N protein (0.5 *µ*g/mL) for 48 h. (**A**) Capillary formation ability was tested by a tube formation assay, and the total length of the capillaries is shown in the graph. (**B**) Cell migration potential was analysed by a wound healing assay. Representative images are shown. Scale bars, 100 μm. The graphs display the means ± S.D. (*n* = 3). *P < 0.05, **P < 0.01, ****P < 0.0001

Then, we investigated the effect of TGF-β neutralization on EndMT markers in SARS-CoV-2-stimulated endothelial cells. In general, neutralizing antibodies abolished the effect of specific SARS-CoV-2 proteins. However, it should be noted that the effect of antibodies correlated with the type of TGF-β stimulated by individual viral proteins. Thus, anti-TGF-β1 diminished the effect of protein S, which, according to our research, stimulates TGF-β1 secretion, and anti-TGF-β2 antibodies inhibited the effect of protein N, which stimulates TGF-β2 secretion (Fig. 6A-C). Moreover, the inhibitory effect of neutralizing antibodies was more significant in the EndMT stimulated by the N protein. This effect was particularly noticeable for mesenchymal markers (Fig. 6A) or contractile proteins (Fig. 6C), in which anti-TGF-β2 antibodies almost completely eliminated the effect of the N protein. We observed convergent results for neutralizing antibodies when studying the behaviour of endothelial cells stimulated with SARS-CoV-2 proteins. We also found that anti-TGF-β1 antibodies inhibited the S protein-dependent reduction of capillary length by 30 and 76% in HUVECs and HMEC-1 cells, respectively, and anti-TGF-β2 inhibited capillary length by 20% in both studied cell lines (Fig. 7A). On the other hand, anti-TGF-β1 inhibited the N protein-dependent reduction of capillary length by only 15% and 19% in HUVECs and HMEC-1 cells, respectively. In comparison, anti-TGF-β2 inhibited capillary length by 66% and 79% in HUVECs and HMEC-1 cells, respectively (Fig. 7A).

A similar effect of neutralizing antibodies on inhibiting cell migration stimulated by SARS-CoV-2 proteins was observed. Since the protein S had a relatively small influence on cell migration, the effects of the anti-TGF-β1 and anti-TGF-β2 antibodies were relatively weak and statistically insignificant (Fig. 7B). However, an apparent inhibitory effect of the antibodies on N protein-stimulated cell migration was observed. Anti-TGF-β1 inhibited the migration of HUVECs and HMEC-1 cells by 44% and 30%, respectively, and anti-TGF-β2 inhibited the migration of HUVECs and HMEC-1 cells by 86% and 79%, respectively (Fig. 7B).

### Inhibition of TGF-β-dependent pathways abolishes EndMT stimulation induced by SARS-CoV-2 proteins

**Fig. 8:**
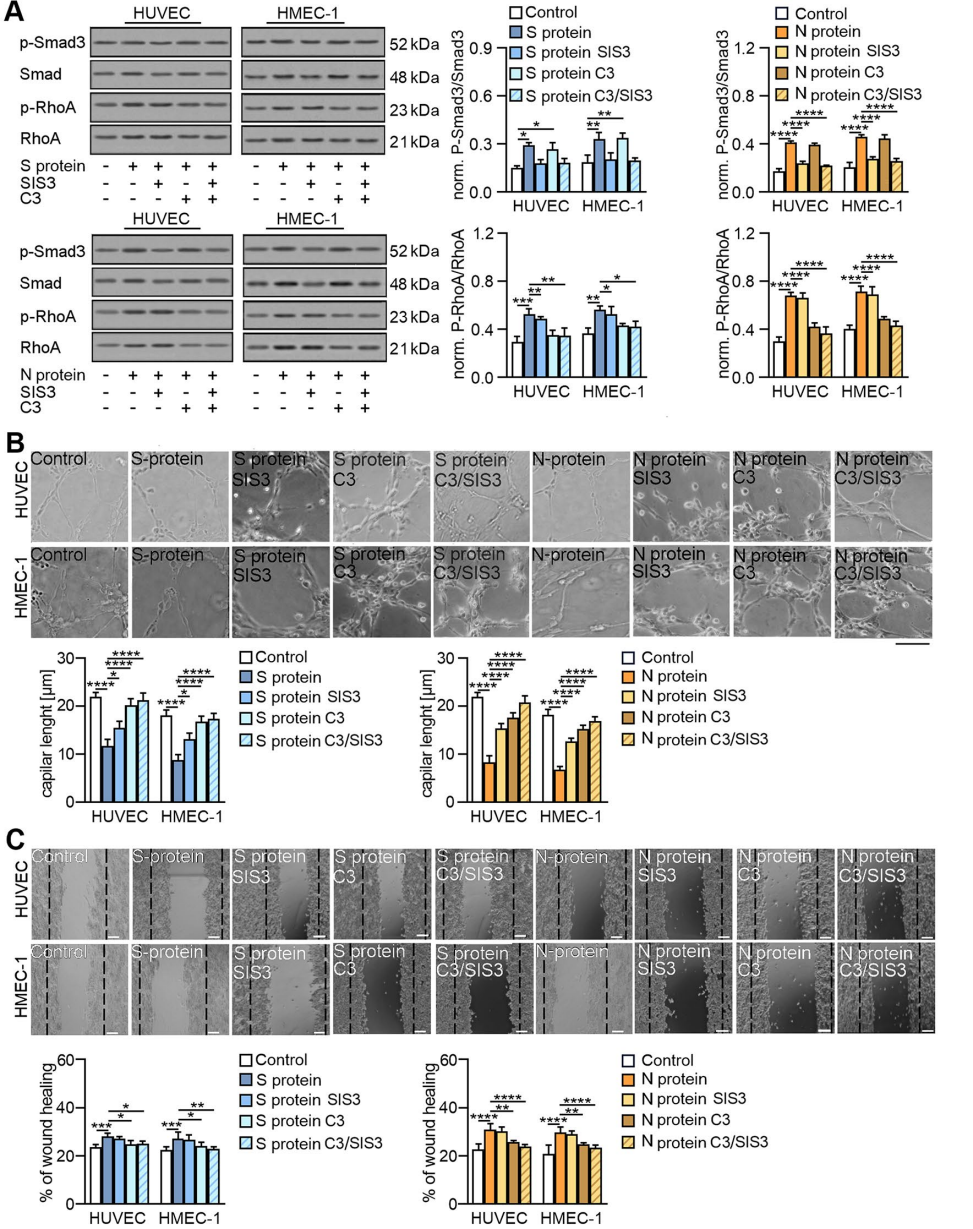
SARS-CoV-2 proteins induce EndMT via TGF-β-dependent pathways. Cells were treated with 3 µM SIS3, 10 µM C3 or both for 1 h prior to stimulation with S protein (0.5 µg/mL) or N protein (0.5 *µ*g/mL) for 48 h. Then, (**A**) the activities of Smad3 and Rho were analysed by Western blot. Representative blots are shown. Phosphorylated Smad3 and RhoA levels were normalized to total Smad3 and RhoA levels, respectively. (**B**) Capillary formation ability was tested by a tube formation assay, and the total length of the capillaries is shown in the graph. (**C**) Cell migration potential was analysed by a wound healing assay. Representative images are shown. Scale bars, 100 μm. The graphs display the means ± S.D. (*n* = 3). *P < 0.05, **P < 0.01, ***P < 0.001, ****P < 0.0001

We showed that TGF-β neutralization blocked SARS-CoV-2-dependent EndMT induction. Thus, in the next step, we investigated whether inhibiting TGF-β-dependent pathways using a Smad3 inhibitor (SIS3) or a RhoA inhibitor (C3 transferase) would also have a similar effect [28]. . First, we analysed whether SARS-CoV-2 proteins activate TGF-β-dependent pathways and whether inhibitors block their signal transduction. Endothelial cells exhibited approximately 1.9- and 2.3-fold higher Smad phosphorylation and about 1.6- and 2-fold higher RhoA phosphorylation after stimulation with the SARS-CoV-2 S and N proteins, respectively (Fig. 8A). We also observed that the inhibitors exhibited specificity of action against relevant signalling proteins (Fig. 8A). Furthermore, when we analysed the effect of Smad and RhoA inhibitors on EndMT, we found that they blocked the transition process of endothelial cells. We noticed that the SIS3 and C3 inhibitors decreased the level of mesenchymal protein markers (Snail, N-cadherin, and vimentin) while increasing the level of endothelial protein markers (claudin and occludin) in SARS-CoV-2-stimulated endothelial cells (Fig. S2A-B). Moreover, the contraction ability of SARS-CoV-2-stimulated cells, as estimated by the protein levels of caldesmon, tropomyosin, and α-SMA, was also diminished by Smad and RhoA inhibitors (Fig. S2C). Furthermore, it is worth emphasizing that the effect of inhibiting EndMT induction by SARS-CoV-2 virus proteins was most significant when both inhibitors were used simultaneously.

Next, we studied whether Smad and RhoA inhibitors restore endothelial cell function by facilitating the formation of a capillary structure and loss of migratory potential. Previously, we observed that the SARS-CoV-2 S and N proteins decreased capillary formation by 40% and 60%, respectively. Treatment with the SIS3 inhibitor blocked the S protein-dependent reduction of capillary length by 38% and 58% in HUVECs and HMEC-1 cells, respectively, and treatment with the C3 inhibitor blocked this effect by 83% in both studied cell lines (Fig. 8B). A higher blocking effect of 92% was observed when both inhibitors were used simultaneously. A similar level of inhibitor action was observed in cells stimulated with the N protein. (Fig. 8B).

Then, we investigated the effect of inhibitors on cell migration stimulated by SARS-CoV-2 proteins. Although the S protein slightly stimulated cell migration, the inhibitors effectively blocked cell migration. When both inhibitors were used together, they inhibited migration by 30% and 10% in HUVECs and HMEC-1 cells, respectively (Fig. 8C). In comparison, the effect of the inhibitors was much more significant in the N protein-treated cells, which were characterized by more remarkable movement ability. SIS3 inhibited the migration of HUVECs and HMEC-1 by 10%, C3 inhibitor by 60%, and SIS3 and C3 together by 89 and 71%, respectively (Fig. 8C).

### SARS-CoV-2 proteins stimulate EndMT through MRTF regulation

**Fig. 9:**
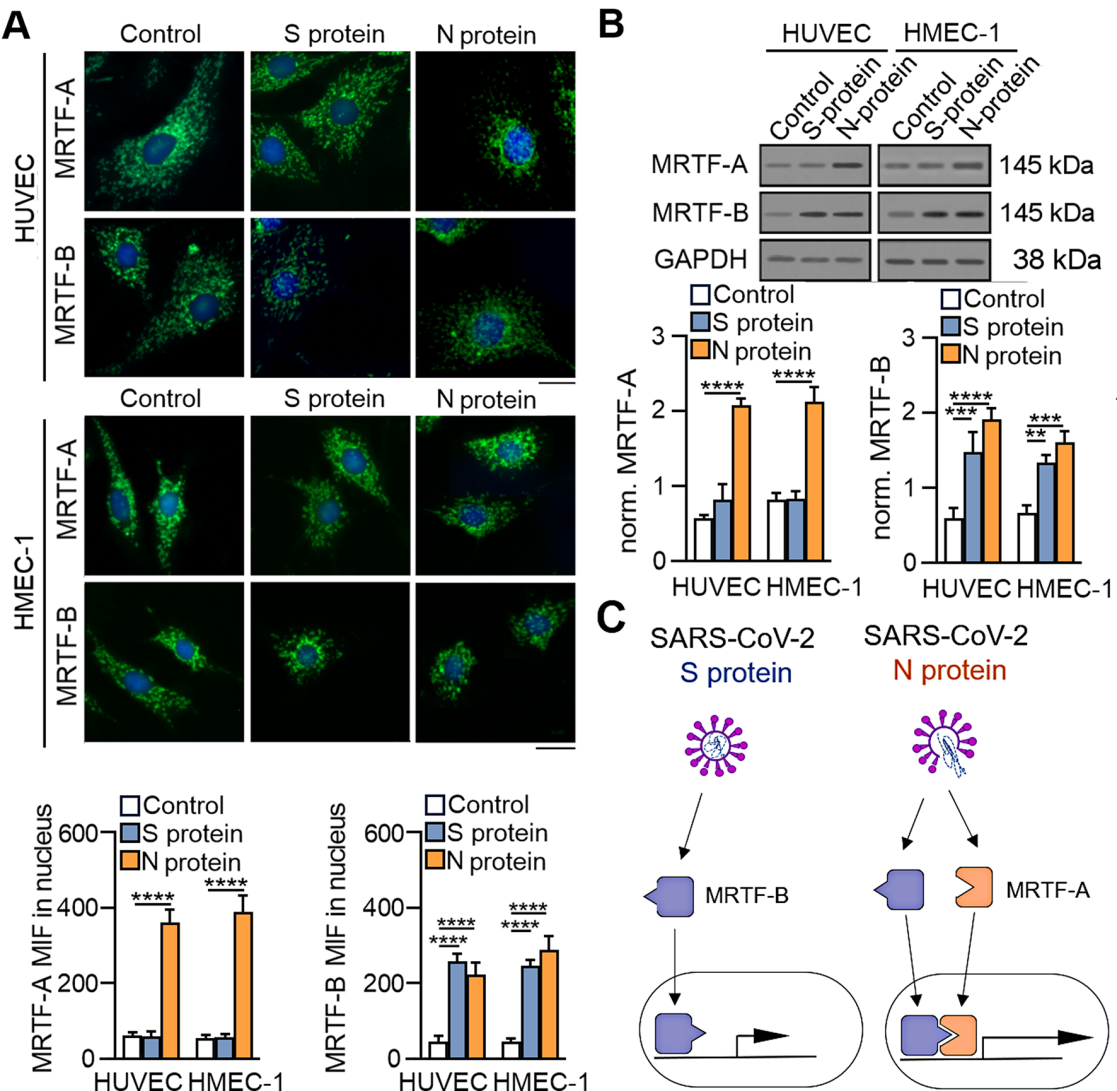
The SARS-CoV-2 protein induces nuclear MRTF translocation and stimulates MRTF expression. Cells were treated with S protein (0.5 *µ*g/mL) or N protein (0.5 *µ*g/mL) for 48 h. Then, (**A**) MRTFA and MRTFB nuclear density were assessed by fluorescence microscopy. Representative images are shown. Scale bars, 100 μm. (**B**) The MRTFA and MRTFB total protein levels were measured by Western blotting. The protein level was normalized to that of GAPDH. Representative blots are shown. The graphs display the means ± S.D. (*n* = 3). *P < 0.05, **P < 0.01, ****P < 0.0001. (**C**) Schematic overview of the role of the SARS-CoV-2 S and N proteins in MRTFs activation

Our previous studies showed that TGF-β family members induce EndMT through various pathways [24]. We revealed that TGF-β2 was responsible for so-called fast EndMT stimulation through MRTF-A and MRTF-B regulation, whereas TGF-β1 was responsible for slow EndMT induction through MRTF-B engagement only. Therefore, in the next step, we investigated whether SARS-CoV-2 S and N proteins, which are specific for stimulating the secretion of different TGF-β family members, would similarly affect individual MRTF proteins. MRTFs act as transcriptional coactivators; therefore, we examined their nuclear translocation and revealed that the S protein induces only MRTF-B translocation and that the N protein induces both MRTF-A and MRTF-B translocation (Fig. 9A). Moreover, we also observed that SARS-CoV-2 proteins stimulate the expression of MRTFs in endothelial cells. We revealed that S protein treatment increased MRTF-B protein levels by 2.43- and 2-fold compared with those in untreated HUVECs and HMEC-1 cells, respectively (Fig. 9B). In contrast, the MRTF-A protein level was only slightly but not significantly increased. Interestingly, we observed that SARS-CoV-2 N protein treatment increased both MRTF protein levels. The MRTF-A protein level increased 2.1- and 1.9-fold, and the MRTF-B protein level increased 2.1- and 1.6-fold in HUVECs and HMEC-1 cells, respectively (Fig. 9B). Thus, we observed that SARS-CoV-2 proteins engaged different MRTFs in EndMT regulation and that the S protein acted through MRTF-B stimulation. In contrast, the N protein acted through MRTF-A and MRTF-B (Fig. 9C).

### SARS-CoV-2 proteins modulate MRTF translocation through TGF-βs

**Fig. 10:**
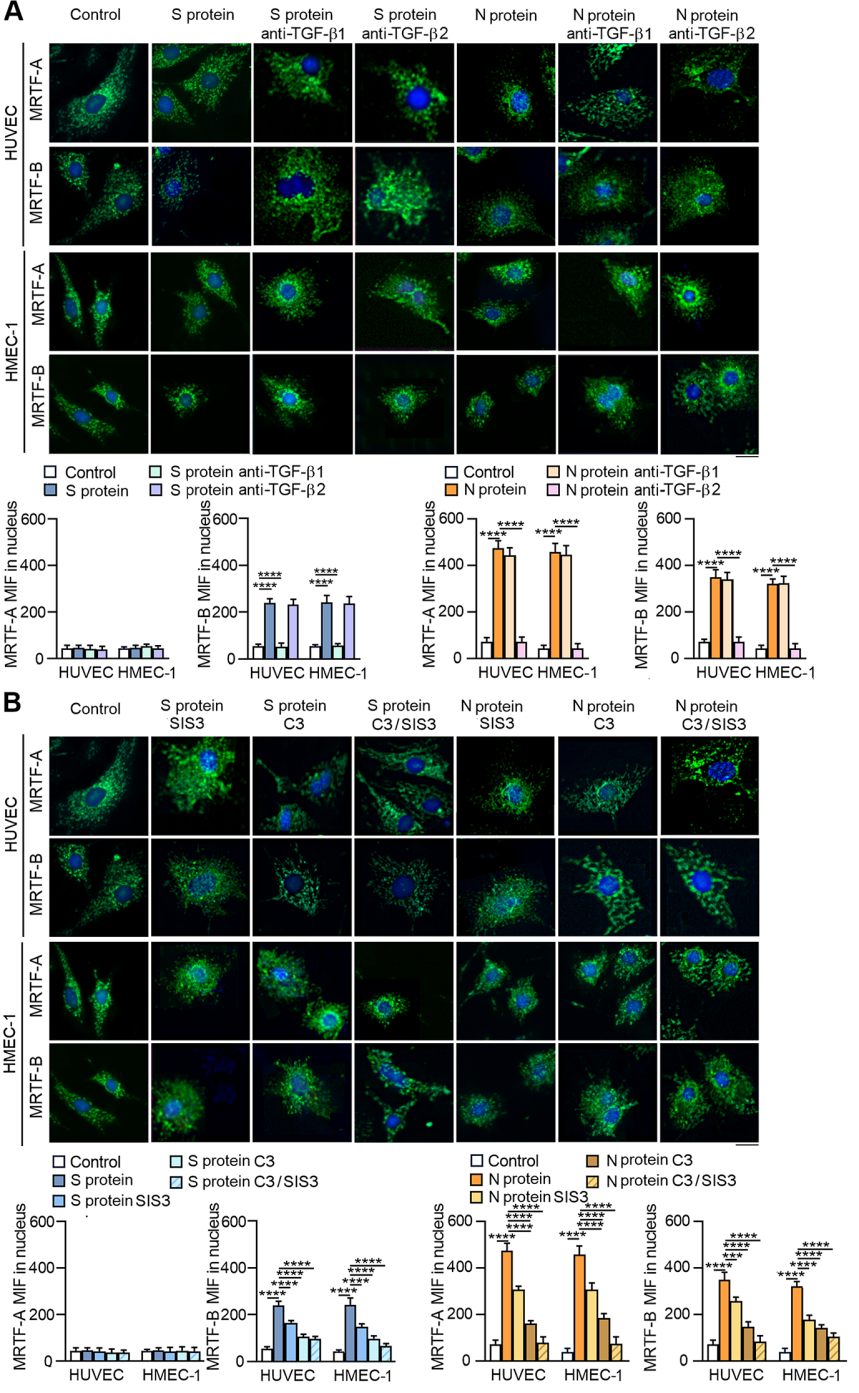
Inhibition of the TGF-β pathway affected SARS-CoV-2-dependent induction of MRTFs. (**A**) Cells were treated with 5 µg/mL anti-TGF-β1 or anti-TGF-β2 antibodies for 1 h prior to stimulation with S protein (0.5 µg/mL) or N protein (0.5 µg/mL) for 48 h. (**B**) Cells were treated with 3 µM SIS3, 10 µM C3 or both for 1 h prior to stimulation with S protein (0.5 µg/mL) or N protein (0.5 *µ*g/mL) for 48 h. Then, the MRTF protein level in the nuclear fraction was assessed by fluorescence microscopy. Representative images are shown. Scale bars, 100 μm. The graphs display the means ± S.D. (*n* = 3). ****P < 0.0001

To confirm that SARS-CoV-2 proteins modulate MRTFs in a TGF-β-dependent manner, we used inhibitors of the TGF-β pathway. First, we utilized TGF-β1- and TGF-β2-neutralizing antibodies and observed that they inhibited MRTF translocation to the nucleus after SARS-CoV-2 stimulation (Fig. 10A). It should be noted that the results obtained were consistent with previous observations regarding the specific effect of SARS-CoV-2 proteins on the secretion of individual TGF-βs and the modulation of particular MRTFs. Anti-TGF-β1 antibodies inhibited the nuclear translocation of MRTF-B in S protein-stimulated cells, whereas anti-TGF-β2 antibodies blocked the nuclear translocation of MRTF-A and MRTF-B in N protein-stimulated cells (Fig. 10A and Fig. S3A).

Since anti-TGF-β antibodies blocked MRTF nuclear translocation, we used Smad3 and RhoA inhibitors to confirm that inhibiting TGF-β-dependent pathways would also have a similar effect. As expected, the Smad3 and RhoA inhibitors blocked the S protein-dependent nuclear translocation of MRTF-B and the N protein-dependent nuclear translocation of both MRTF proteins (Fig. 10B and Fig. S3B). When both inhibitors were used together, the translocation of MRTF proteins into the nucleus was blocked entirely. The same changes were also observed when examining the effect of anti-TGF-β antibodies on the total protein level of MRTFs (Fig. S4A-B).

### Aspirin might both prevent and reverse SARS-CoV-2-induced EndMT

**Fig. 11:**
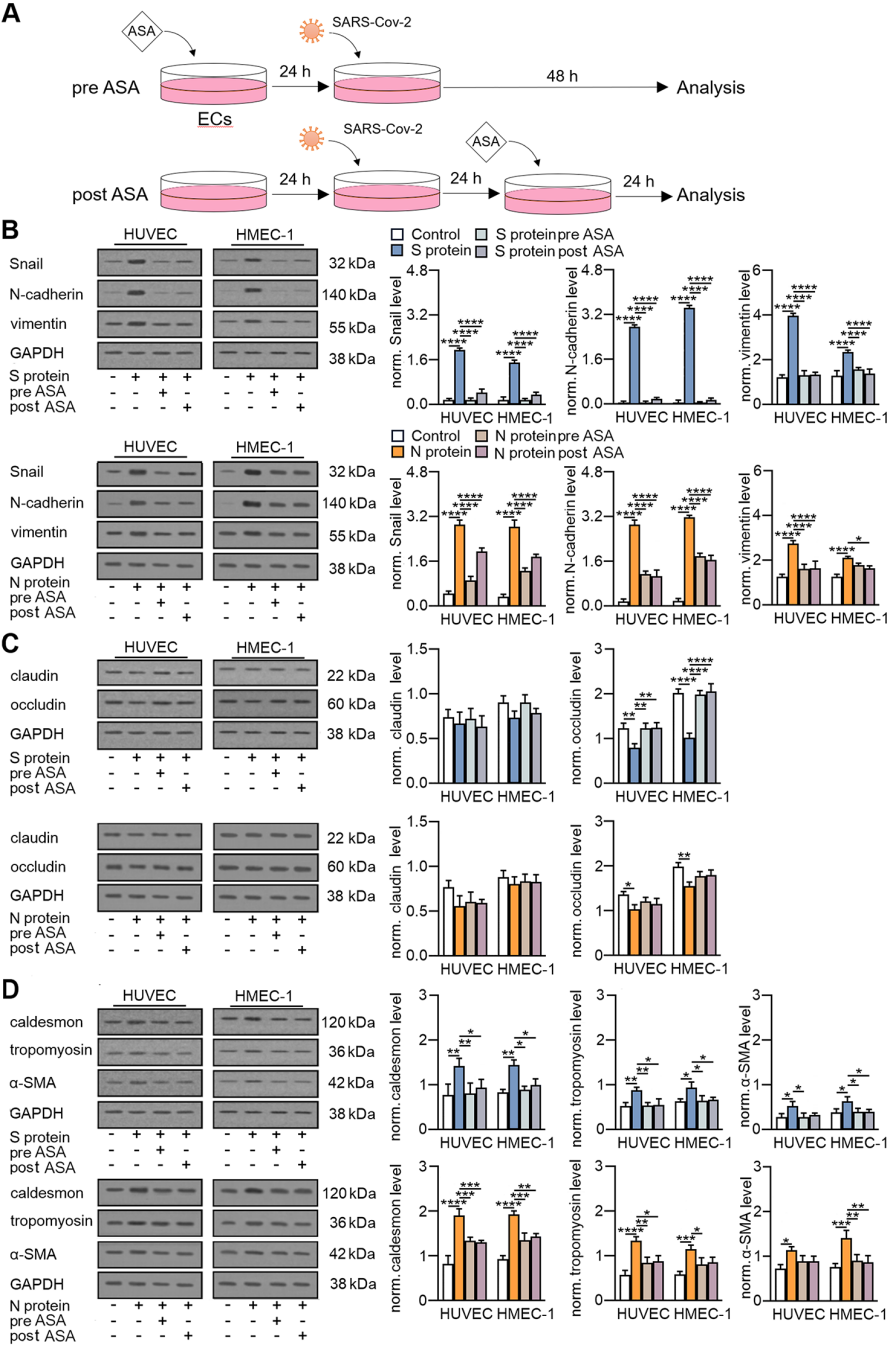
Aspirin (ASA) prevents SARS-CoV-2 EndMT stimulation. (**A**) Schematic overview of the use of ASA in endothelial cells stimulated with SARS-CoV-2 proteins. Cells were treated with ASA (2.5 mM) 24 h prior to or 24 h after stimulation with SARS-CoV-2 proteins. 48 h after treatment with the SAR-CoV-2 proteins, the cells were subjected to further analysis. (**B**) The levels of mesenchymal markers, (**C**) the levels of endothelial markers and (**D**) the levels of contraction protein markers were analysed by Western blot. Representative blots are shown. The protein level was normalized to GAPDH. The graphs display the means ± S.D. (*n* = 3). *P < 0.05, **P < 0.01, ***P < 0.001, ****P < 0.0001

Previously, we demonstrated that aspirin (ASA) could inhibit vincristine-dependent EndMT induction [29]. Herein, we analysed whether ASA might inhibit EndMT stimulated by SARS-CoV-2 proteins. Moreover, since patients are often diagnosed long after SARS-CoV-2 infection, we wondered whether ASA could reverse the EndMT already induced by SARS-CoV-2. Therefore, we explored the effect of ASA on preventing and reversing the EndMT induced by SARS-CoV-2 proteins (Fig. 11A).

First, we analysed whether ASA could restore the levels of mesenchymal and endothelial markers in SARS-CoV-2-stimulated cells. We observed that ASA diminished the SAR-CoV-2-dependent increase in mesenchymal protein levels. ASA reduced the Snail, N-cadherin, and vimentin proteins to basal levels and by approximately 50% in cells stimulated with the SARS-CoV-2 S and N protein, respectively (Fig. 11B). Moreover, we revealed that ASA only partially restored endothelial protein levels to the state prior to cell treatment with SARS-CoV-2. A statistically significant effect of ASA was observed only for occludin, the protein level of which returned to the level before treatment with the S protein (Fig. 11C).

We also explored the effect of ASA on caldesmon, tropomyosin, and α-SMA protein levels modulated by SARS-CoV-2 treatment. We observed that ASA completely abolished the stimulatory effect of SARS-CoV-2 on contractile proteins in cells stimulated with the S protein and inhibited the increase in protein levels by 50% in cells stimulated with N (Fig. 11D).

**Fig. 12:**
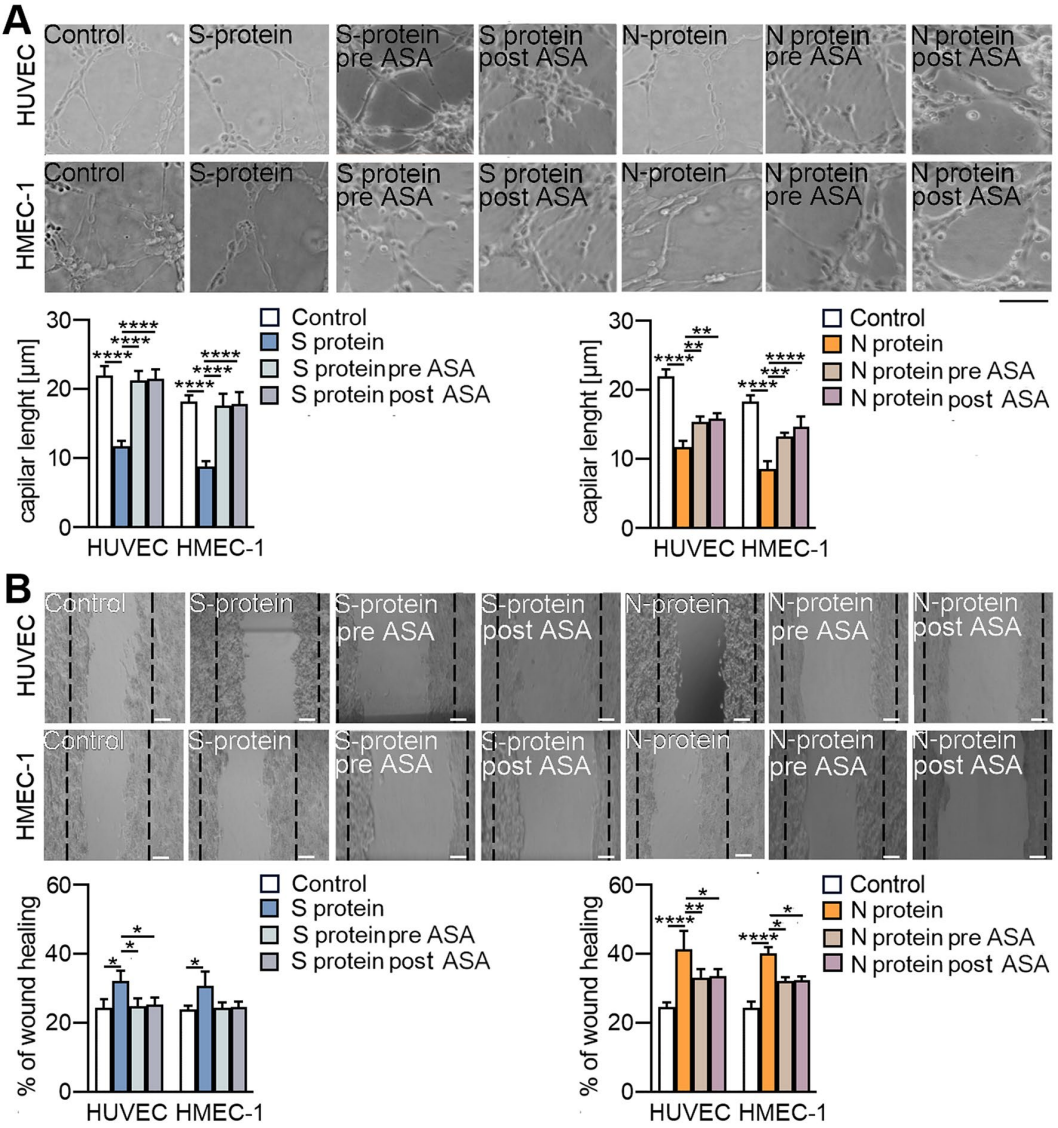
Aspirin restores the loss of endothelial cell behaviour mediated by SARS-CoV-2 stimulation. Cells were treated with ASA (2.5 mM) 24 h prior to or 24 h after stimulation with SARS-CoV-2 proteins. 48 h after treatment with the SAR-CoV-2 proteins, the cells were subjected to behavioural analysis. (**A**) Capillary formation ability was studied by tube formation assays, and the total length of capillaries is shown in the graph. (**B**) Cell migration potential was analysed by a wound healing assay. Representative images are shown. Scale bars, 100 μm. The graphs display the means ± S.D. (*n* = 3). *P < 0.05, **P < 0.01, ****P < 0.0001

Next, we investigated the effect of ASA on the behaviour of endothelial cells stimulated with SARS-CoV-2 proteins. ASA completely abolished the S protein-dependent reduction of capillary length effect in both studied cell lines and inhibited the reduction of capillary length induced by the N protein by 60% and 40% in HUVECs and HMEC-1 cells, respectively (Fig. 12A). A coincident effect of ASA on the inhibition of cell migration stimulated by SARS-CoV-2 proteins was observed. We revealed that ASA eliminated the cell migration stimulated by the S protein and inhibited the movement of N protein-stimulated cells by 50% in both studied cell lines (Fig. 12B). Notably, ASA prevented and reversed the SARS-CoV-2-dependent changes in endothelial behaviour at the same level (Fig. 12A-B).

### Aspirin abrogates SARS-CoV-2-dependent EndMT through dysregulation of the TGF-β-MRTF axis

**Fig. 13:**
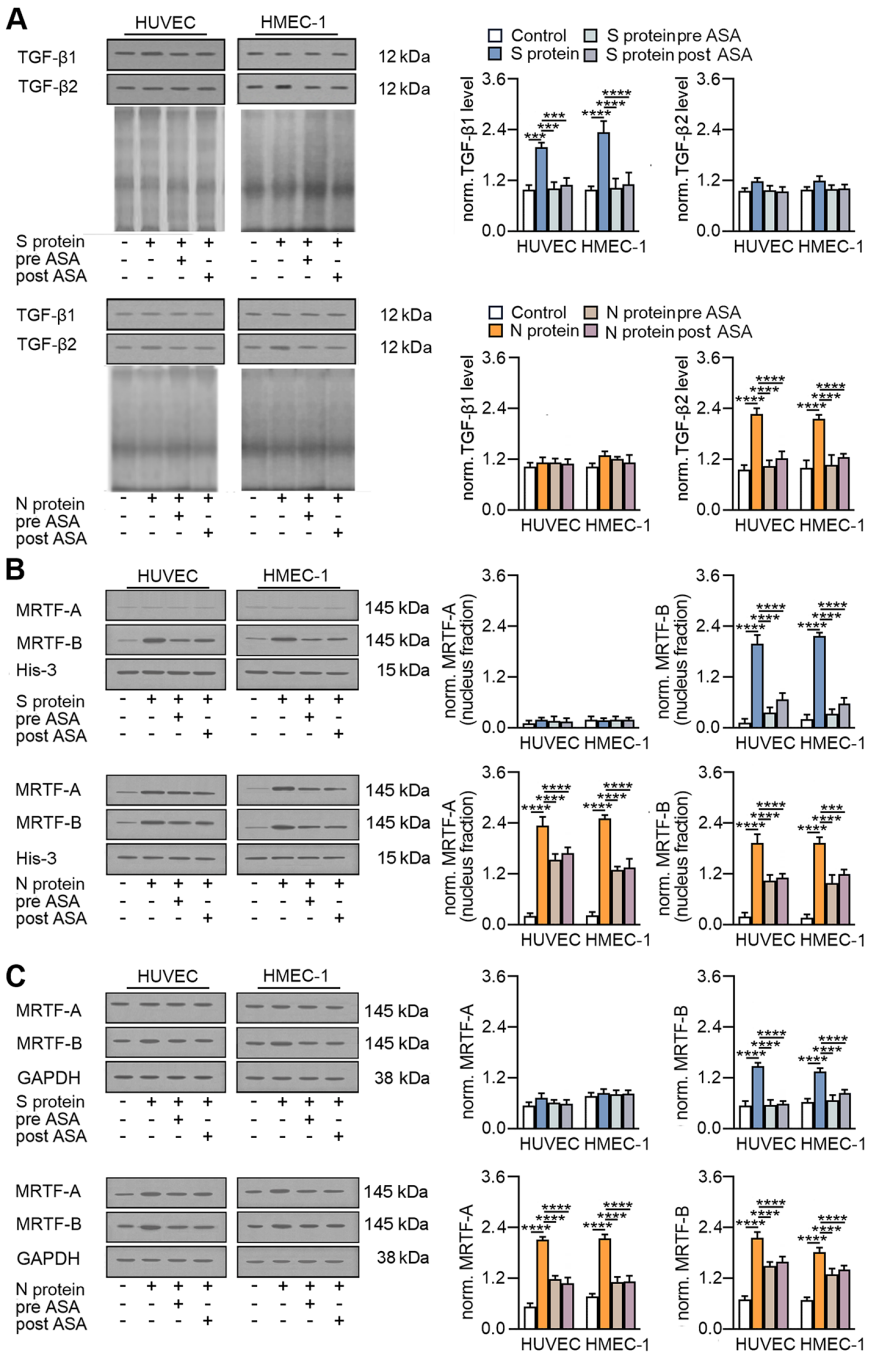
Aspirin disturbs the SARS-CoV-2-dependent TGF-β-MRTF pathway axis. Cells were treated with ASA (2.5 mM) 24 h prior to or 24 h after stimulation with SARS-CoV-2 proteins. 48 h after treatment with the SAR-CoV-2 proteins, cells were subjected to Western blot analysis. (**A**) The levels of TGF-β1 and TGF-β2 were measured by Western blotting. Coomassie blue was used as the loading control. Representative blots are shown. The graphs display the means ± S.D. (*n* = 3). *P < 0.05, ***P < 0.001, ****P < 0.0001. The MRTF (**B**) protein level in the nuclear fraction and (**C**) total protein level was measured by Western blot. Representative blots are shown. Protein levels were normalized to His-3 (nuclear fraction) or GAPDH (total protein). The graphs display the means ± S.D. *n* = 3). ***P < 0.001, ****P < 0.0001

Since we previously showed that aspirin inhibited vincristine-dependent EndMT stimulation by blocking the secretion of TGF-βs [29], we verified whether ASA inhibited the ability of the SARS-CoV-2 protein to stimulate EndMT in this same manner. We revealed that ASA treatment completely inhibited TGF-β1 and TGF-β2 secretion stimulated by the SARS-CoV-2 S and N proteins, respectively (Fig. 13A). Furthermore, we investigated the effect of ASA on the modulation of MRTFs and observed that aspirin inhibited the nuclear translocation of MRTF-A stimulated by the S protein almost to the basal level (Fig. 13B). However, the inhibitory effect of aspirin on MRTF-A and MRTF-B translocation stimulated by the N protein was lower, approximately 50% for both MRTFs (Fig. 13B). Finally, we investigated the influence of ASA on MRTF expression in cells stimulated with SARS-CoV-2 proteins. We noticed that the changes in the expression of MRTF proteins in cells stimulated with SARS-CoV-2 proteins due to the action of ASA were analogous to those observed in the modulation of MRTF transport to the nucleus (Fig. 13C).

## Discussion

SARS-CoV-2 is a type of virus that belongs to the coronavirus family and is responsible for causing the acute respiratory disease COVID-19. Although the name indicates the respiratory system as the main site of infection, the SARS-CoV-2 virus can also attack other organs in the human body. The course of the disease itself may be mild; however, it often also leads to inflammation and, consequently, acute respiratory syndrome. In this phase, the production of pro-inflammatory cytokines rapidly increases, which can induce fibrosis and lead to severe lung damage [30]. A long-term inflammation might induce fibrotic signalling, which can result in multiple-organ failure and, consequently, death. Apart from pulmonary fibrosis [31], SARS-CoV-2 infection can also cause fibrosis in other organs and affect kidney, heart and liver function [32–34].

Fibrosis is a slow-developing condition that can eventually lead to tissue degeneration and, consequently, to diseases of various organs, including the lungs, liver, intestines, heart, skin and kidneys [35]. Under these pathological conditions, there is excessive accumulation of fibrous connective tissue in the area of the extracellular matrix (ECM) of injured tissues. When tissue becomes extremely fibrotic, there are persistent healing problems that lead to abnormal functioning of organs or tissues. A key cellular mediator of fibrosis is myofibroblasts, which arise from various sources, including endothelial cells, in a process called endothelial-mesenchymal transition (EndMT) [28]. Endothelial cells, like epithelial cells, are characterized by high surface angiotensin-converting enzyme 2 (ACE2) expression. Since ACE2 is the primary entry mechanism for SARS-CoV-2 [18], the endothelium is also a crucial gateway to viral infection. It has been shown that SARS-CoV-2 binds to ACE2 via Spike proteins (S proteins), leading to virus endocytosis [36]. This process reduces the ACE2 level and increases the Ang II level, which upregulates the expression of the profibrotic growth factor TGF-β. It has been proposed that the SARS-CoV-2 infection drives EndMT, which might induce pulmonary fibrosis [14, 16]. However, the exact molecular mechanism of SARS-CoV-2-dependent EndMT modulation has not yet been investigated.

In this study, TGF-β-stimulated endothelial cells (HMEC-1 cells and HUVECs) that undergo EndMT were previously characterized [24] and were used as a reference model. As a model of SARS-CoV-2 infection, we employed cells stimulated with the recombinant Spike (S protein) and Nucleocapsid (N protein), the main SARS-CoV-2 proteins known to play a significant role in coronavirus entry into cells and the life cycle [37, 38]. We employed a concentration of SARS-CoV-2 proteins used in previous studies [39] and close to achievable in patients with severe COVID-19 [40, 41].

We were the first to observe that stimulation of endothelial cells with the SARS-CoV-2 Spike and Nucleocapsid proteins stimulates endothelial cells, which leads to EndMT. This was confirmed by an increase in mesenchymal markers and contractile proteins and a decrease in endothelial proteins. The observation was coincident with previous showing changing of EndMT markers in histopathological micrographs of lung samples from COVID-19 patients [42]. Furthermore, we observed that the modulations within the EndMT protein markers were accompanied by the acquisition of mesenchymal cell behaviour, manifesting as the inhibition of capillary-like structure formation and the increased cell motility. Our data confirm and extend previous observations on tissues from COVID-19 patients showing changes in endothelial morphology, such as destruction, damage to intercellular junctions or contact disruption with the basement membrane [43, 44]. However, it is essential to note that our study, for the first time, showed that the potency of EndMT induction varied depending on the type of SARS-CoV-2 protein. We found that the Nucleocapsid protein induced EndMT more potently than the Spike protein. Therefore, it seems that the effectiveness of EndMT induction and, consequently, the potential development of fibrosis may be related to the phase of virus infection of endothelial cells. Since the Spike protein is necessary for SARS-CoV-2 entry process [18], we considered that the phase of initial virus interaction with endothelial cells should not induce far-reaching changes in the functioning of the endothelium. On the other hand, the Nucleocapsid protein, which plays an essential role in virus replication, particle assembly, and release [45], can be considered a marker of the advanced phase of virus interaction with endothelial cells and, according to our findings, might cause severe endothelial dysregulation.

Severe COVID-19 is accompanied by a massive release of inflammatory mediators, which may cause acute respiratory distress syndrome. Consequently, excessive secretion of pro-inflammatory cytokines occurs, leading to systemic inflammation and cytokine storms that threaten the patient’s life [30]. In severe COVID-19, the immune response is at least partially modulated by TGF-β [46, 47]. The secretion of this crucial EndMT modulator [24] might be regulated by SARS-CoV-2 proteins [48]. Herein, we confirmed that SARS-CoV-2 might stimulate endothelial cells to secrete TGF-β. By employing inhibitors of Smad- and non-Smad-dependent pathways, we further demonstrated that SARS-CoV-2 proteins induce EndMT in a TGF-β-dependent manner. However, we observed a clear difference in the modulation of TGF-β following stimulation with individual SARS-CoV-2 proteins. The Spike protein induced TGF-β1 secretion, while Nucleocapsid TGF-β2 only. To confirm that the Spike-TGF-β1- and Nucleocapsid-TGF-β2-dependent effects on endothelial cell stimulation occurred, neutralizing antibodies were used. We revealed that the reduction the access of active TGF-β1 and TGF-β2 molecules significantly suppressed the EndMT stimulated by the Spike and the Nucleocapsid, respectively.

Herein, we proved that SARS-CoV-2 infection induced EndMT, which was previously postulated by others [14, 16]. Furthermore, we also found that SARS-CoV-2 proteins might stimulate endothelial cells via different TGF-β-dependent molecular pathways. The Spike protein acts through TGF-β1, while the Nucleocapsid protein engages TGF-β2, which stimulates EndMT more effectively. This raises the question of why individual SARS-CoV-2 proteins have such selective effects. First, we investigated why SARS-CoV-2 proteins stimulate the secretion of various TGF-β family members. It has been widely reported that the main route of SARS-CoV-2 entry into cells is through interactions between the Spike protein and ACE-2 [18]. Hamming et al. showed that ACE-2 is widely present in humane epithelial and endothelial cells [49]; thus, the SARS-CoV-2 virus might easily spread through the body and infect critical organs. During virus infection, ACE-2 is downregulated, which contributes to impaired endothelial function [50]. Our results are consistent with this, as we found that the Spike protein reduced the surface expression of the ACE-2 protein, while the Nucleocapsid protein did not affect it. Moreover, several studies investigating the interaction between TGFβ and ACE-2 revealed that loss of ACE-2 enhances TGF-β1 secretion [51, 52]. This observation suggested that Spike-dependent TGF-β1 activation is based on ACE-2 downregulation. To explain the effect of Nucleocapsid on TGF-β2 secretion, we focused on an alternative, less explored mechanism by which SARS-CoV-2 interacts with Toll-like receptor 4 (TLR4) [53, 54]. TLR4 is a pattern recognition receptor that regulates inflammation during viral and bacterial infections [54]. It has been shown that TLR4 activation induces pro-inflammatory responses through ROS generation [27, 55]. It has been proposed recently that the SARS-CoV-2 Spike protein might directly interact with TLR4 [56, 57]. However, we observed only a weak interaction of the Spike protein with TLR4, manifested by a slight increase in surface TLR4 levels and ROS generation. In contrast, Quian et al. showed that the Spike protein was ineffective in endothelial cell activation even at doses two times higher doses than those we utilized [58]. On the other hand, we revealed clear activation of TLR4 by Nucleocapsid protein. This was confirmed by a significant increase in TLR4 levels on the cell surface and ROS production, which was blocked by a TLR4 inhibitor. It seems that ROS generated by Nucleocapsid-stimulated cells might act as a positive loop and elevate TLR4 levels. This finding is consistent with previous research showing that oxidative stress increases TLR4 surface expression [59]. Additionally, it has recently been suggested that Nucleocapsid might activate the Smad3-dependent pathway [46, 60], which could also induce ROS production [61]. Previously, we revealed that oxidative stress stimulates TGFβ2 but not TGFβ1 secretion during the EndMT process [62]. Therefore, we propose that the N protein stimulates TGF-β2 secretion through the TLR4-Smad-ROS axis. This assumption requires the extracellular presence and action of the Nucleocapsid protein, which generally resides within SARS-CoV-2 virions or inside the host cell during infection. However, Li et al. showed that the Nucleocapsid protein is detectable in the serum of 76% of SARS-CoV-2 patients [63]. This indicates that the N protein could be released to the extracellular niche during virus-dependent host cell lysis.

Next, we conducted a study to understand why different proteins of the same virus might cause the same process but with varying intensities. Our findings suggest that SARS-CoV-2 triggers EndMT through TGF-β1 or TGF-β2 secretion via the action of the Spike protein and Nucleocapsid, respectively. Moreover, since the N protein induced EndMT with greater potency than the S protein, we concluded that TGF-β2 is a more effective EndMT inducer than TGF-β1. This finding is consistent with our previous studies on EndMT modulation, in which we proposed a new model for regulating the expression of Snail, the main transcription factor responsible for EndMT [24]. We observed that TGF-β1 controls Snail expression through MRTF-B, while TGF-β2 alters both MRTF-A and MRTF-B, reinforcing Snail expression and promoting EndMT more extensively. Herein, we found that the Spike protein stimulates only MRTF-B in a TGF-β1-dependent manner, whereas the Nucleocapsid through TGF-β2, engages both MRTF-A and MRTF-B. Thus, the SARS-CoV-2 Nucleocapsid protein is more potent at stimulating EndMT, and it seems to play a more crucial role in the course of COVID-19 than the Spike protein. These findings are consistent with previously reported correlations between high concentrations of IgG against the Nucleocapsid protein and poor outcomes in COVID-19 patients [64–66]. Moreover, other studies have shown that TLR4 expression, which, according to the results of our study, is activated mainly by Nucleocapsid, is correlated with severe COVID-19 [54].

Other studies indicated also that nonsteroidal anti-inflammatory drugs (NSAIDs) might help in faster recovery and better prognosis for SARS-CoV-2-infected patients [67, 68]. Aspirin with anti-inflammatory and anti-thrombotic properties, seems to be a promising protector against severe COVID-19 [69, 70]. However, other studies suggest that the evidence supporting NSAIDs use during SARS-CoV-2 infection remains fragile and premature, and particular caution should be exercised during NSAIDs administration in COVID-19 patients [71]. Since we previously showed that aspirin could inhibit EndMT [29], we here investigated whether aspirin would also have anti-EndMT effects stimulated by SARS-CoV-2 and, consequently, act as an anti-fibrotic agent. Generally, handling SARS-CoV-2 is based on preventing infection or reducing its negative effects within the COVID-19 course. Therefore, we investigated the potential of aspirin both in preventing the effects of virus infection and in reversing changes already induced by SARS-CoV-2. We showed that aspirin is equally effective at inhibiting EndMT and reversing changes that occur in SARS-CoV-2-stimulated endothelial cells. Aspirin completely protects cells against EndMT changes caused by the Spike protein and partially protects against the harmful effects of the Nucleocapsid protein. In both cases, we observed a dramatic downregulation of TGF-β-MRTF interaction. This is consistent with previous evidence revealing that aspirin might attenuate fibrosis by suppressing the TGF-β signalling pathway [72, 73]. Therefore, we propose that aspirin treatment might reduce tissue fibrosis development in patients with COVID-19 through dysregulation of the EndMT process.

## Conclusions

**Fig. 14:**
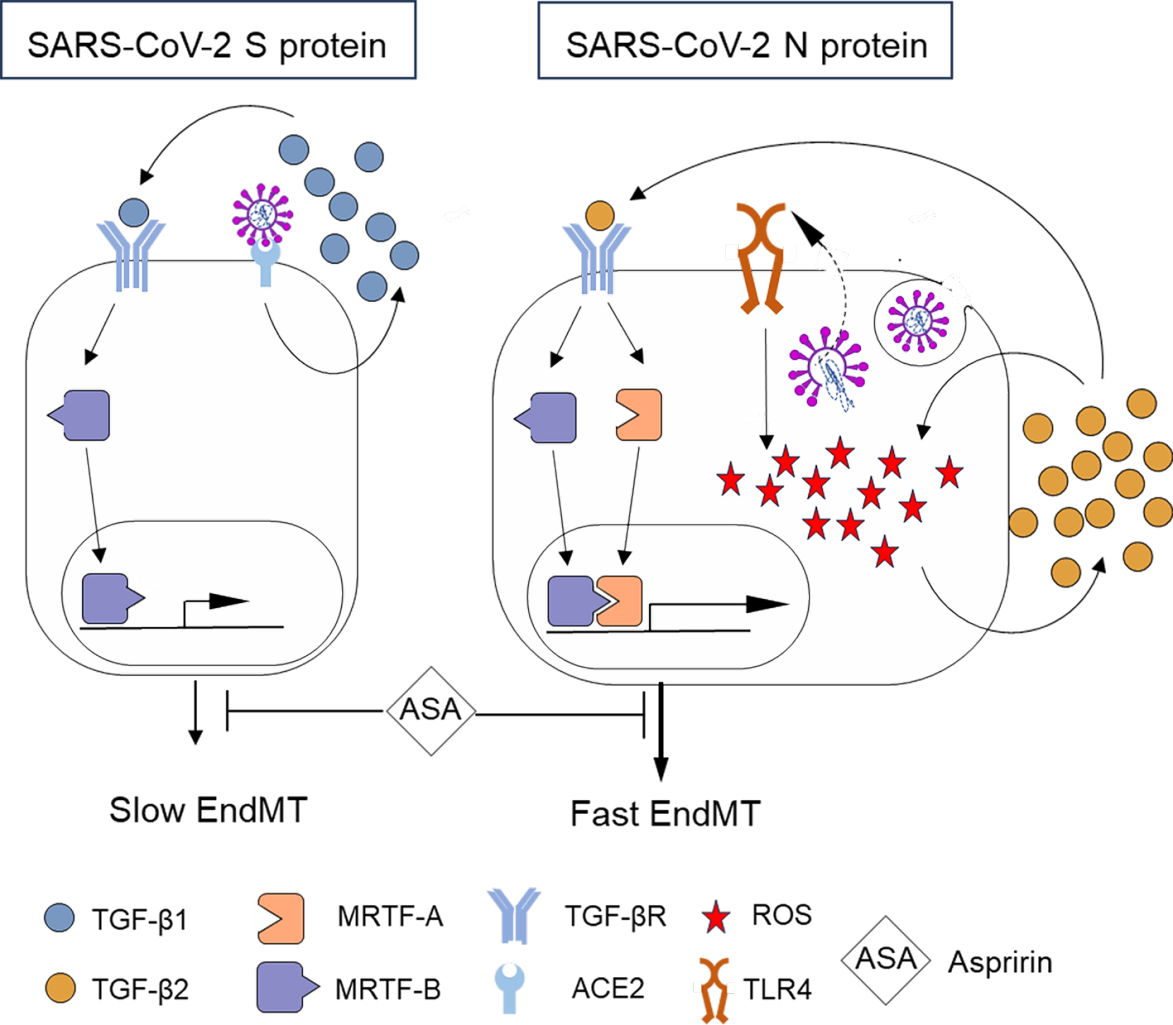
Scheme of SARS-CoV-2-dependent EndMT induction. The SARS-CoV-2 S protein induces TGF-β1 production through interaction with ACE2. Secreted TGF-β1 activates TGF-βR and stimulates MRTF-B nuclear translocation, which, in turn, induces slow EndMT. In contrast, the SARS-CoV-2 N protein induces TLR4 surface expression, which stimulates ROS production and, in turn, TGF-β2 secretion. TGF-β2 activates TGF-βR and promotes MRTF-A and MRTF-B nuclear translocation and, in turn, fast EndMT induction. Moreover, the stimulation of the EndMT process caused by both proteins of the SARS-CoV-2 virus can be inhibited by aspirin (ASA).

Our research demonstrated that the SARS-CoV-2 Spike and Nucleocapsid proteins can activate endothelial cells, which leads to EndMT and potentially the development of fibrosis. We revealed that the Nucleocapsid protein is more potent in EndMT stimulation than the Spike protein. We have gained insights into this process and revealed that Nucleocapsid-dependent EndMT induction is based on TLR4 stimulation, ROS production, and, consequently, TGF-β2 secretion. TGF-β2 then activates the translocation of MRTF-A and MRTF-B into the nucleus, leading to significant expression of the Snail protein and rapid induction of EndMT. In contrast, the mechanisms underlying the Spike-dependent EndMT induction involve a reduction in ACE-2 and increased TGF-β1 secretion. The released TGF-β1 induces nuclear translocation of only the MRTF-B protein, which has a weaker effect on the increase in Snail protein expression and, consequently, stimulates the less potent EndMT. Figure 14 shows a potential signalling pathway for SARS-CoV-2-induced EndMT.

Furthermore, we provide evidence that aspirin has the potential to modulate EndMT induced by SARS-CoV-2 proteins. We demonstrated that aspirin can both inhibit and reverse changes in endothelial cells stimulated with SARS-CoV-2 proteins. Aspirin completely removed the effect of the Spike protein on endothelial cells, while the effect of the stronger stimulant, the Nucleocapsid protein of SARS-CoV-2, was partially suppressed. This study enhances our understanding of SARS-CoV-2-mediated pathological changes in the endothelium and highlights the potential of aspirin as a therapeutic drug for fibrosis in COVID-19 patients.

### Electronic supplementary material

Below is the link to the electronic supplementary material.

Supplementary Material 1

## Data Availability

The datasets used and/or analysed during the current study are available from the corresponding author on reasonable request.
