# Correction: Diverse roles of SARS-CoV-2 spike and nucleocapsid proteins in EndMT stimulation through the TGF-β-MRTF axis inhibited by aspirin

**DOI:** 10.1186/s12964-024-01766-9

**Published:** 2024-07-29

**Authors:** Wojciech M. Ciszewski, Lucyna A. Woźniak, Katarzyna Sobierajska

**Affiliations:** 1 Department of Molecular Cell Mechanisms, Medical University of Lodz, Str. 6/8, Lodz 92-215, Mazowiecka, Poland; 2 Department of Structural Biology, Medical University of Lodz, Str. 7/9, Lodz 90-752, Mazowiecka, Poland

**Correction: Cell Commun Signal 22**,** 296 (2024)**.

10.1186/s12964-024-01665-z

Following publication of the original article [1], it was noticed that there was an error in the funding information. The correct grant number should be 2017/26/D/NZ7/00633.

The incorrect funding statement:

This research was supported by a grant funded by the National Science Centre, Cracow, Poland, grant number 2017/01/X/NZ7/00499 and the statutory funds of the Medical University of Lodz.

The correct funding statement:

This research was supported by a grant funded by the National Science Centre, Cracow, Poland, grant number 2017/26/D/NZ7/00633 and the statutory funds of the Medical University of Lodz.

## References

[1] Ciszewski WM, Woźniak LA, Sobierajska K. Diverse roles of SARS-CoV-2 spike and nucleocapsid proteins in EndMT stimulation through the TGF-β-MRTF axis inhibited by aspirin. Cell Commun Signal. 2024;22:296. 10.1186/s12964-024-01665-z. 38807115 10.1186/s12964-024-01665-z PMC11134719

