# Supplementary material for: Diverse roles of SARS-CoV-2 Spike and Nucleocapsid proteins in EndMT stimulation through the TGF-β-MRTF axis inhibited by aspirin
Source: Cell Commun Signal. 2024 May 28;22:296. doi: 10.1186/s12964-024-01665-z (PMC11134719; doi:10.1186/s12964-024-01665-z)
Supplement: Supplementary file 1 — Supplementary Material 1 [file 12964_2024_1665_MOESM1_ESM.docx]

**Diverse role of SARS-CoV-2 Spike and Nucleocapsid proteins in EndMT stimulation through TGF-β-MRTFs axis inhibited by aspirin**

Wojciech M. Ciszewski^1^, Lucyna A. Woźniak^2^, Katarzyna Sobierajska^1,^*

^1^Department of Molecular Cell Mechanisms, Medical University of Lodz, Mazowiecka 6/8, 92-215 Lodz, Poland

^2^Department of Structural Biology, Medical University of Lodz, Żeligowskiego 7/9, 90-752 Lodz, Poland

*Author to whom correspondence should be addressed.


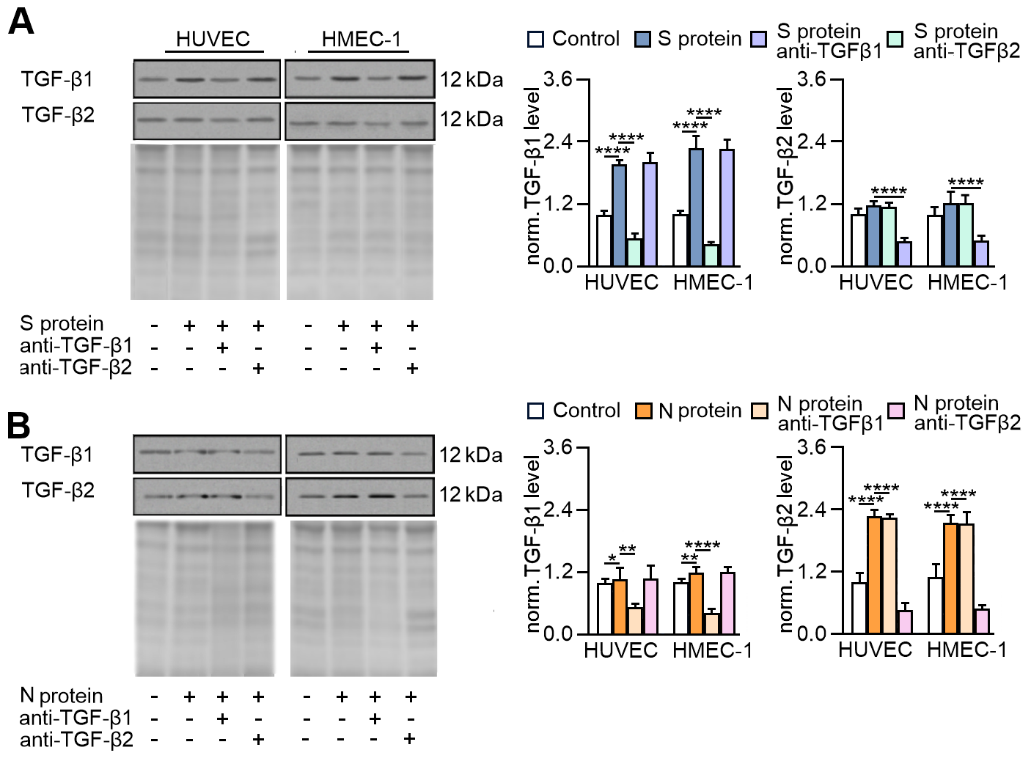


**Supplementary Fig. 1**. SARS-CoV-2 protein stimulate TGF-β1 and TGF-β2 secretion in endothelial cells. (**A**) Cells were treated with S-protein (0.5 *μ*g/mL) or N-protein (0.5 *μ*g/mL) for 48 hours. Then, the level of TGF-β1 or TGF-β2 was measured by Western blot. (**B**) Cells were treated with 5 µg/mL of anti-TGF-β1 or anti-TGF-β2 antibodies for 1 hour prior to stimulation with SARS-CoV-2 proteins as before. The level of TGF-β1 or TGF-β2 was measured by Western blot. As the loading control, Coomassie blue was presented. The representative blots are shown. The graphs display means ± S.D. (n=3). *P< 0.05, **P< 0.01, ****P< 0.0001.


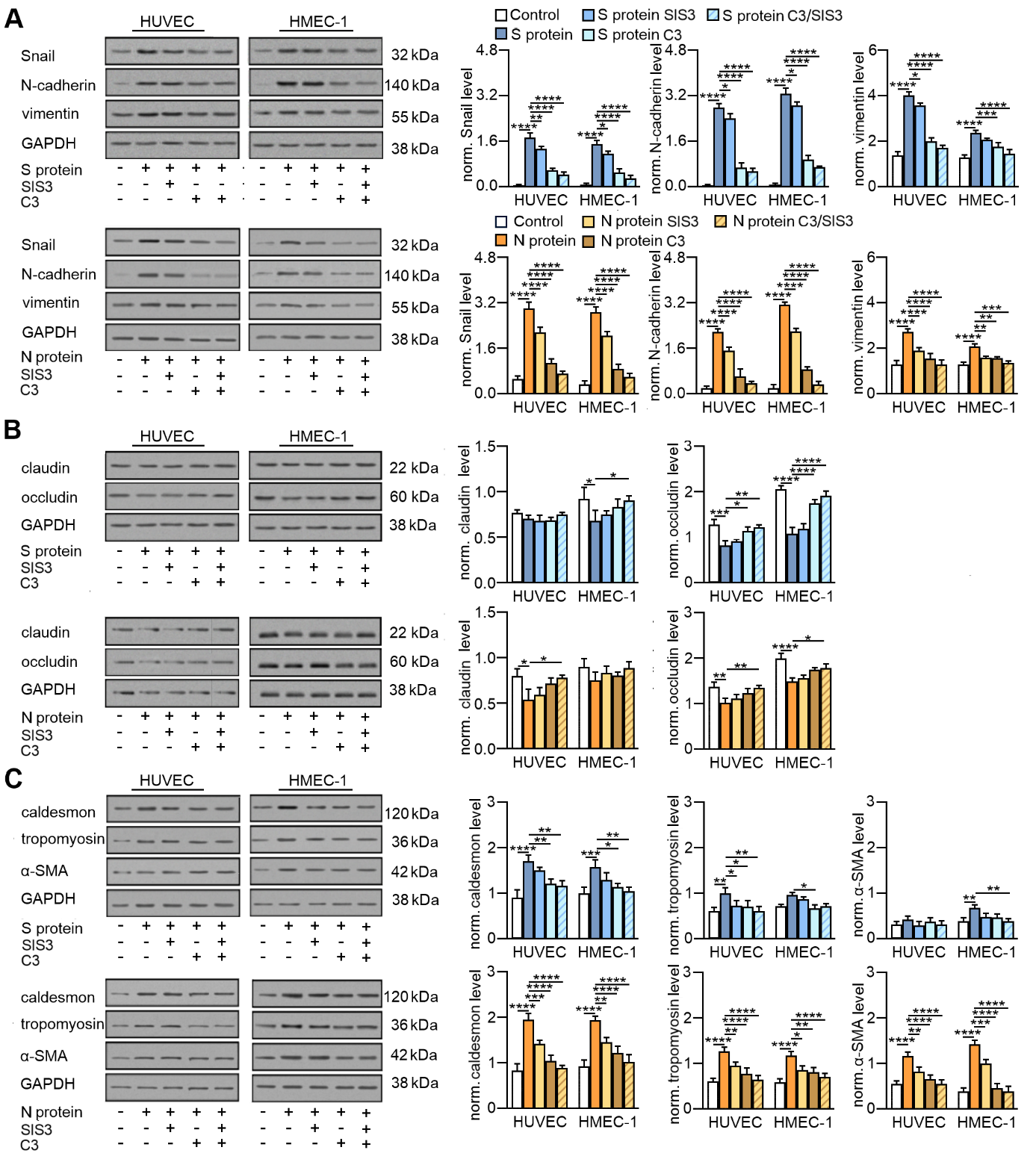


**Supplementary Fig. 2**. SARS-CoV-2 proteins induce EndMT via TGF-βs-dependent pathways. Cells were treated with 3 µM SIS3 or 10 µM C3 or mixed both for 1 h prior to stimulation with S protein (0.5 μg/mL) or N protein (0.5 μg/mL) for 48 hours. Then, (A) The levels of mesenchymal markers, (B) the levels of endothelial markers and (C) the level of contraction protein markers were analysed by Western blot assay. The representative blots are shown. Protein level was normalized to GAPDH. The graphs display means ± S.D. (n=3). *P< 0.05, **P< 0.01, ****P< 0.0001.


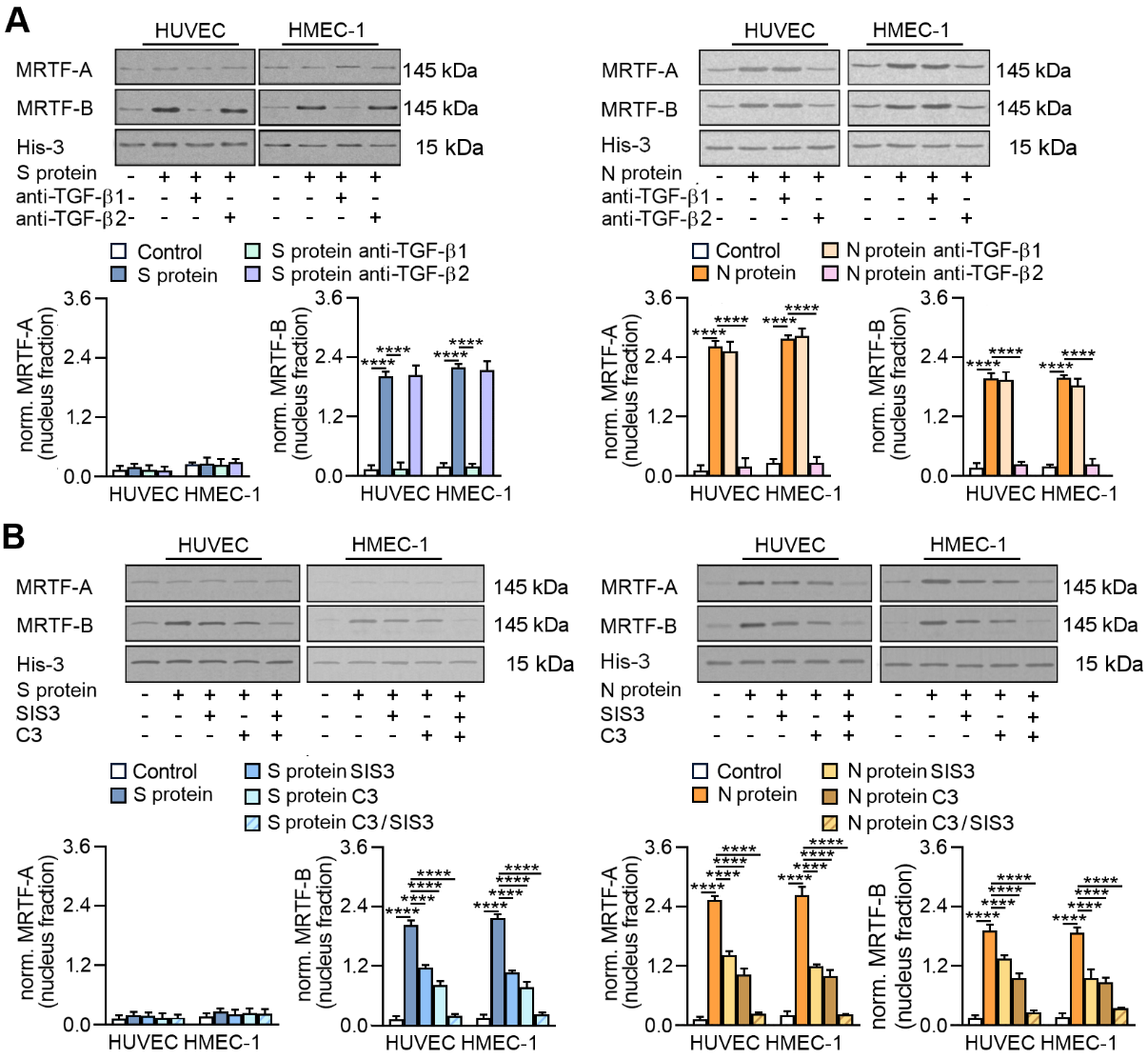


**Supplementary Fig. 3** Inhibition of TGF-βs pathways affected SARS-CoV-2-dependent induction of MRTFs nuclear translocation. (**A**) Cells were treated with 5 µg/mL of anti-TGF-β1 or anti-TGF-β2 antibodies for 1 hour prior to stimulation with S protein (0.5 μg/mL) or N protein (0.5 μg/mL) for 48 hours. (**B**) Cells were treated with 3 µM SIS3 or 10 µM C3 or mixed both for 1 h prior to stimulation with S protein (0.5 μg/mL) or N protein (0.5 *μ*g/mL) for 48 hours. Then, MRTFs protein level in nuclear fraction was measured by Western blot. Protein level was normalized to His-3. The representative blots are shown. The graphs display means ± S.D. (n=3). ****P< 0.0001.


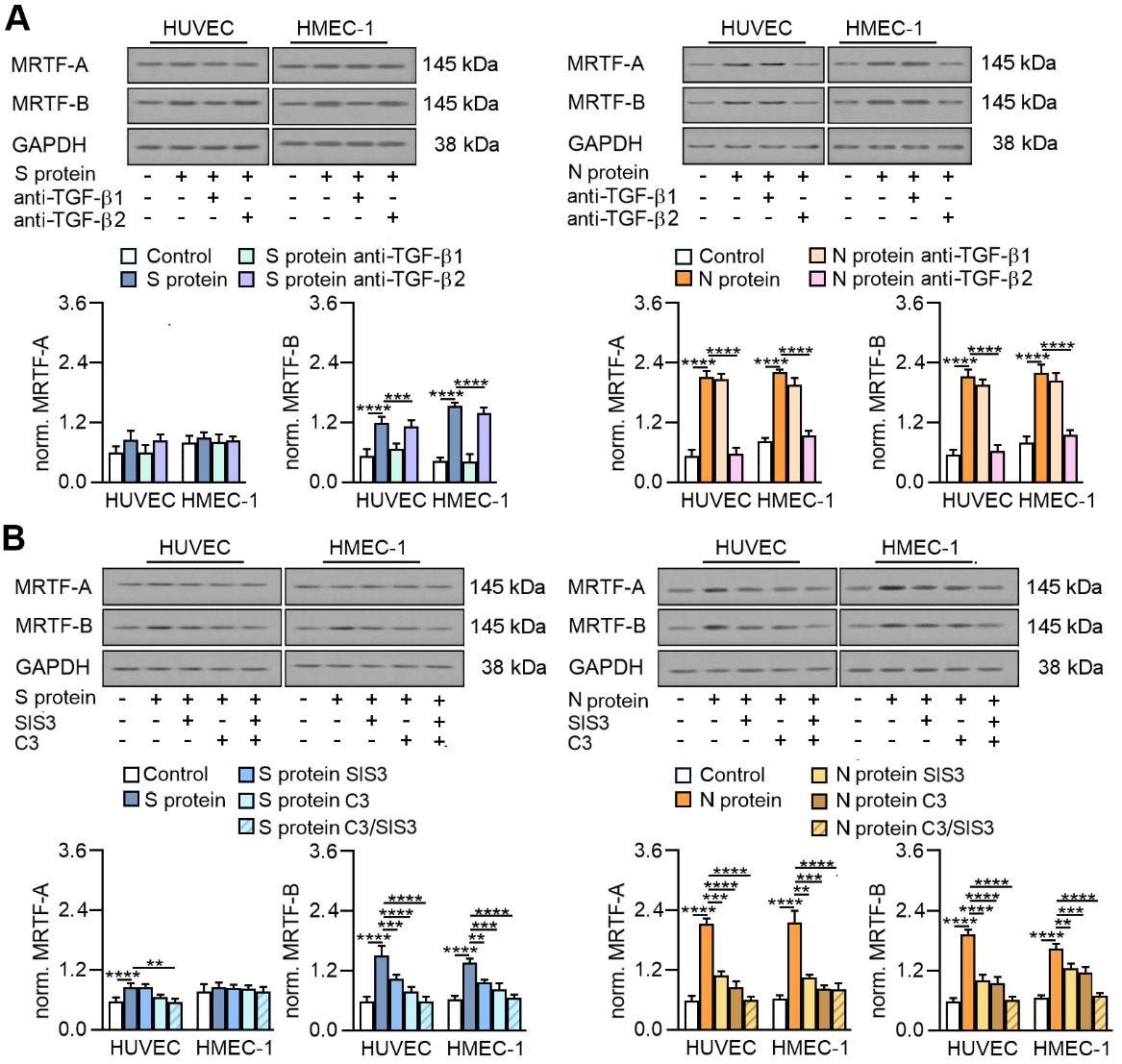


**Supplementary Fig. 4** Inhibition of TGF-βs pathways affected SARS-CoV-2-dependent induction of MRTFs expression. (**A**) Cells were treated with 5 µg/mL of anti-TGF-β1 or anti-TGF-β2 antibodies for 1 hour prior to stimulation with S protein (0.5 μg/mL) or N protein (0.5 μg/mL) for 48 hours. (**B**) Cells were treated with 3 µM SIS3 or 10 µM C3 or mixed both for 1 h prior to stimulation with S protein (0.5 μg/mL) or N protein (0.5 *μ*g/mL) for 48 hours. Then, total MRTFs protein level was measured by Western blot. Protein level was normalized to GAPDH. The representative blots are shown. The graphs display means ± S.D. (n=3). ****P< 0.0001.
